# LAG3 regulates antibody responses in a murine model of kidney transplantation

**DOI:** 10.1172/JCI172988

**Published:** 2025-05-13

**Authors:** Michael Nicosia, Ran Fan, Juyeun Lee, Gabriella All, Victoria Gorbacheva, José I. Valenzuela, Yosuke Yamamoto, Ashley Beavers, Nina Dvorina, William M. Baldwin, Eduardo Chuluyan, Motoo Araki, Brian T. Gaudette, Robert L. Fairchild, Booki Min, Anna Valujskikh

**Affiliations:** 1 Department of Inflammation and Immunity and; 2 Department of Cardiovascular and Metabolic Sciences, Lerner Research Institute, Cleveland Clinic, Cleveland, Ohio, USA.; 3 Universidad de Buenos Aires, Consejo Nacional de Investigaciones Científicas y Técnicas, Centro de Estudios Farmacológicos y Botánicos (CEFYBO), Facultad de Medicina, Buenos Aires, Argentina.; 4 Universidad de Buenos Aires, Facultad de Medicina, Departamento de Microbiología, Parasitología e Inmunología, Buenos Aires, Argentina.; 5 Department of Urology, Okayama University Graduate School of Medicine, Dentistry and Pharmaceutical Sciences, Okayama, Japan.

**Keywords:** Immunology, Transplantation, Adaptive immunity

## Abstract

Lymphocyte activation gene 3 (LAG3) is a coinhibitory receptor expressed by various immune cells. Although the immunomodulatory potential of LAG3 is being explored in cancer and autoimmunity, there is no information on its role after organ transplantation. Our study investigated the functions of LAG3 in a mouse model of renal allograft rejection. LAG3^–/–^ recipients rapidly rejected MHC-mismatched renal allografts that were spontaneously accepted by WT recipients, with graft histology characteristic of antibody-mediated rejection. Depletion of recipient B cells but not CD8^+^ T cells significantly extended kidney allograft survival in LAG3^–/–^ recipients. Treatment of WT recipients with an antagonistic LAG3 antibody enhanced anti-donor immune responses and induced kidney damage associated with chronic rejection. The studies of conditional LAG3^–/–^ recipients and mixed bone marrow chimeras demonstrated that LAG3 expression on either T or B cells is sufficient to regulate anti-donor humoral immunity but not to induce acute allograft rejection. The numbers and proinflammatory functions of graft-infiltrating NK cells were markedly increased in LAG3^–/–^ recipients, suggesting that LAG3 also regulates the effector stage of antibody-mediated rejection. These findings identified LAG3 as a regulator of immune responses to kidney allografts and a potential therapeutic target for antibody-mediated rejection prevention and treatment.

## Abstract 2

Our studies have identified LAG3 as a novel regulator of humoral alloimmune responses following kidney transplantation

## Graphical abstract

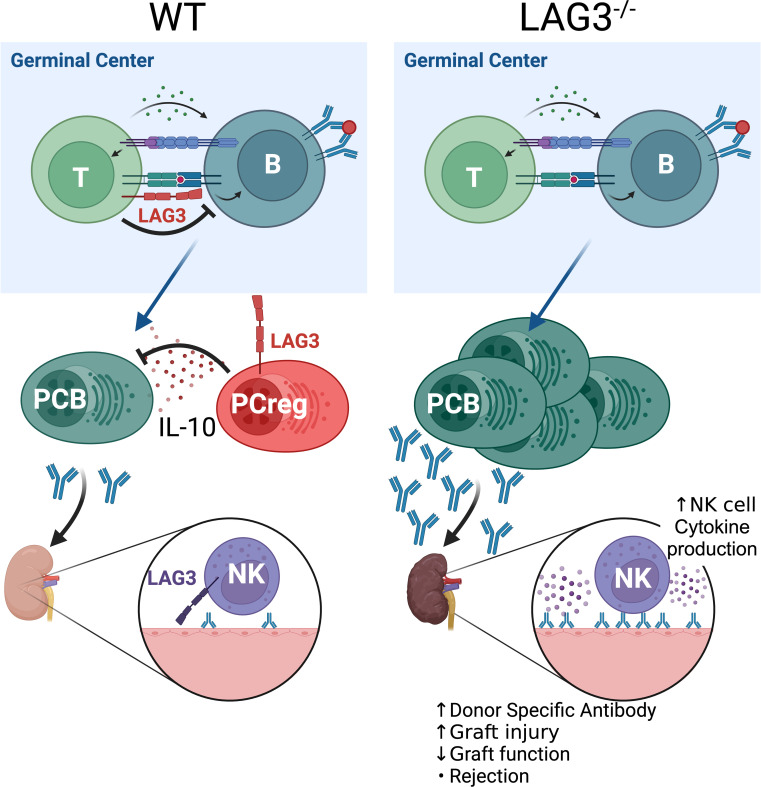

## Introduction

Lymphocyte activation gene 3 (LAG3, CD223) is an immune checkpoint inhibitor of the immunoglobulin superfamily, closely related to CD4 (1). LAG3 is expressed on a number of immune cells including CD4^+^ and CD8^+^ T cells, Tregs, B lymphocytes (1–5), plasmacytoid dendritic cells (6), and NK cells (1). Similar to other coinhibitory receptors such as CTLA4 or PD-1, LAG3 is not expressed by naive T cells, but its expression is induced on CD4^+^ and CD8^+^ T cells following TCR engagement (1). Murine LAG3 shares 69.9% sequence homology with the human protein, preserving structure and function and largely mirrors the expression profile of human LAG3 (7, 8). Analogous to CD4, LAG3 binds to MHC-II molecules on antigen presenting cells (APCs) (9, 10) and additionally interacts with other ligands such as galectin-3 (11), liver sinusoidal endothelial cell lectin (12), a-synuclein fibrils (13), and fibrinogen-like protein 1 (14).

In T cells, LAG3 is colocalized with T cell receptor/CD3 complex within immune synapses and inhibits TCR signaling, leading to impaired cell activation, proliferation, differentiation, and effector functions in the context of autoimmunity, infection, and tumor (15–20). Recent studies have started to reveal the intricacies that underly the suppressive mechanism of LAG3 in T cells (21–23). In addition, LAG3 plays an essential role in Tregs (5, 24, 25), although the data conflict depending on the model and setting (26).

In contrast, there is a paucity of studies investigating the role of LAG3 in B lymphocytes. Activation of murine splenocytes in vitro induces the endogenous expression of LAG3 on CD19^+^ B cells in a T cell– and soluble factor–dependent manner (2). However, the functional consequences of LAG3 upregulation in B cells during humoral immune responses remain to be investigated. LAG3 expression has been shown to be a defining feature of an IL-10–producing subset of plasma cells (27). Although it has been demonstrated that these cells arise from B cell receptor–dependent (BCR-dependent) signals and that their IL-10 production is dependent on innate signals such as TLRs, the role of LAG3 expression on this B cell subset is still poorly understood.

Even though the relative importance of LAG3 on conventional T cells, Tregs, B cells and other cell types during immune responses remains to be determined in different settings, it is a molecule of great interest for clinical interventions. Several mAbs targeting LAG3 with either antagonistic, depleting, or agonistic activities have been developed and are currently in clinical trials for patients with cancer and autoimmune illnesses (28–35). However, LAG3 is not commonly considered as a therapeutic target in organ transplantation, largely due to the paucity of information on the role of this pathway in experimental animal models. Lucas et al. (36) reported that LAG3 blockade prevents tolerance induction in alloreactive CD8^+^ T cells in a mouse model of allogeneic bone marrow transplantation, but that this effect did not depend on LAG3 expression by CD8^+^ T lymphocytes themselves. In a different study, the depletion of LAG3^+^ T cells with mAb extended rat cardiac allograft survival, yet prevented tolerance induction via donor-specific cell transfusion (37). These findings suggest LAG3 involvement in different aspects of allograft rejection and tolerance. Whereas the functions of CTLA4 and PD1 coinhibitory molecules in transplantation have been extensively studied, it is unclear whether and how LAG3 regulates alloimmune responses to solid organ transplants.

In the current study, we investigated the contribution of LAG3 to alloimmune responses using a mouse model of life-supporting renal transplantation and found that recipient LAG3 deficiency resulted in rapid rejection of fully MHC-mismatched renal allografts that were spontaneously accepted by WT recipients. Although LAG3^–/–^ recipients developed increased cellular and humoral anti-donor immune responses, the renal graft tissue injury was characteristic of antibody-mediated rejection and was significantly diminished by the depletion of B cells but not of CD8^+^ T lymphocytes. Analogous increases in IgG antibody responses were observed after immunization of LAG3^–/–^ mice with either T cell–dependent or T cell–independent model antigens, whereas LAG3 cross-linking inhibited antibody secretion by plasma cells in vitro, indicating the B cell–intrinsic role of the LAG3 pathway. Nevertheless, although T cell– or B cell–specific LAG3 conditional knockout recipients and mixed bone marrow chimeras with LAG3 deficiency in both T and B cells had increased donor-specific alloantibody (DSA) production, they did not reject renal allografts, suggesting that LAG3 additionally regulates antibody-mediated allograft injury. In support of this finding, the numbers and proinflammatory functions of graft-infiltrating NK cells, important mediators of antibody-mediated injury, were markedly increased in LAG3^–/–^ allograft recipients compared with WT controls. These data present the first evidence to our knowledge for LAG3 involvement in regulation of humoral alloimmune responses and identify LAG3 as a potential target for diminishing pathogenic alloimmunity.

## Results

### Mice deficient in LAG3 have elevated heterologous alloimmune responses prior to transplantation.

**Figure 1:**
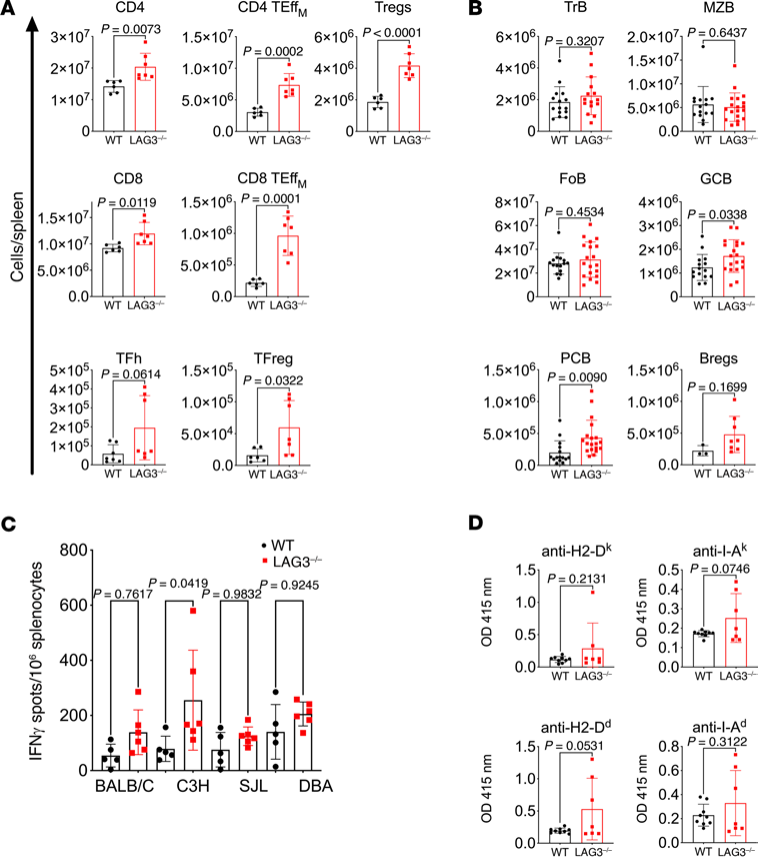
LAG3-deficient mice had expanded lymphocyte subsets and a modest increase in alloreactivity. Naive nontransplanted B6.WT and B6.LAG3^–/–^ mice were euthanized at 10 weeks of age. (**A**) Splenic T cell composition: CD4 (CD3^+^CD4^+^), CD8 (CD3^+^CD8^+^), Tregs (CD3^+^CD4^+^Foxp3^+^), CD4 TEff_M_ (CD3^+^CD4^+^CD62L^lo^CD44^hi^), CD8 TEff_M_ (CD3^+^CD8^+^CD62L^lo^CD44^hi^), TFh (TCRb^+^CD4^+^FoxP3^-^PD-1^+^CXCR5^+^), TFreg (TCRb^+^CD4^+^FoxP3^+^PD-1^+^CXCR5^+^). (**B**) Composition of splenic B cell subsets: FoB (B220^+^IgM^int^CD21/35^int^), MZB (B220^+^IgM^hi^CD21/35^hi^), TrB (B220^+^IgM^hi^CD21/35^lo^), Bregs (CD19^+^CD1d^hi^CD5^+^), GCB (B220^+^GL7^+^CD38^lo^), PC (B220^-^CD138^hi^). (**C**) ELISPOT quantification of the frequencies of alloreactive IFN-γ–secreting splenic T cells in naive nontransplanted B6.WT and B6.LAG3^–/–^ mice against BALB/c, C3H, SJL, and DBA stimulator cells. (**D**) ELISA of serum IgG reactive to allogenic MHC-I and MHC-II molecules. The data represent at least 2 pooled experiments where each symbol represents an individual mouse. Analysis of ELISPOT data utilized 1-way ANOVA with Tukey’s multiple-comparison test, all others used Student’s *t* tests.

Given that LAG3 was demonstrated to play a critical role in immune tolerance, we initially evaluated the immune cells from naive nontransplanted 10-week-old B6.WT and B6.LAG3^–/–^ mice. LAG3 deficiency resulted in a modest increase in splenic cellularity (data not shown) and in the increased numbers of total and effector memory (TEff_M_) CD4^+^ and CD8^+^ T cells, as well as Tregs, compared with WT mice. In addition, mice deficient in LAG3 had elevated numbers of T follicular helper (TFh) and T follicular regulatory (TFreg) cells in the spleen (Figure 1A and Supplemental Figure 1; supplemental material available online with this article; https://doi.org/10.1172/JCI172988DS1). The numbers of major spleen B cell subsets — follicular (FoB), marginal zone (MZB), and transitional (TrB) B cells — were not significantly different between WT and LAG3^–/–^ mice. Notably, mice deficient in LAG3 had significantly increased numbers of germinal center B cells (GCBs) and plasma cells in the spleen, suggesting ongoing B cell activation (Figure 1B and Supplemental Figure 2). There was a trend toward an increase in numbers of regulatory B cells (Bregs) defined as CD19^+^CD1d^hi^CD5^+^ cells. It should be noted that despite the observed shifts toward activated immune cell phenotypes, unmanipulated LAG3^–/–^ mice did not exhibit signs of autoimmune disease up to 6 months of age.

To assess the impact of this phenotypic shift on alloimmunity, we probed both B6.WT and B6.LAG3^–/–^ for T cell reactivity against a panel of allogeneic strains (Figure 1C). An IFN-γ ELISPOT assay demonstrated that compared with WT mice, naive LAG3^–/–^ mice had increased frequencies of preexisting memory T cells reactive against C3H (H2-D^k^) alloantigens. The numbers of BALB/c (H-2^d^) SJL (H-2D^s^)–reactive T cells but not DBA (H2-D^q^)–reactive T cells were elevated in some nontransplanted LAG3^–/–^ mice, but these increases were not statistically significant (Figure 1C). We also tested for the presence of alloreactive antibodies in the sera of nontransplanted mice (Figure 1D). Some of the tested naive LAG3^–/–^ mice (H2-D^b^) contained elevated levels of IgG antibodies against class I and class II MHC molecules from BALB/c (H2-D^d^ I-A^d^) and C3H mice (H2-D^k^ I-A^k^). These results suggest that LAG3 is an important regulator of immune cell homeostasis and as such may play a crucial role during immune responses to transplanted organs.

### Recipient LAG3 deficiency enhances anti-donor alloimmune responses and elicits kidney allograft rejection.

**Figure 2:**
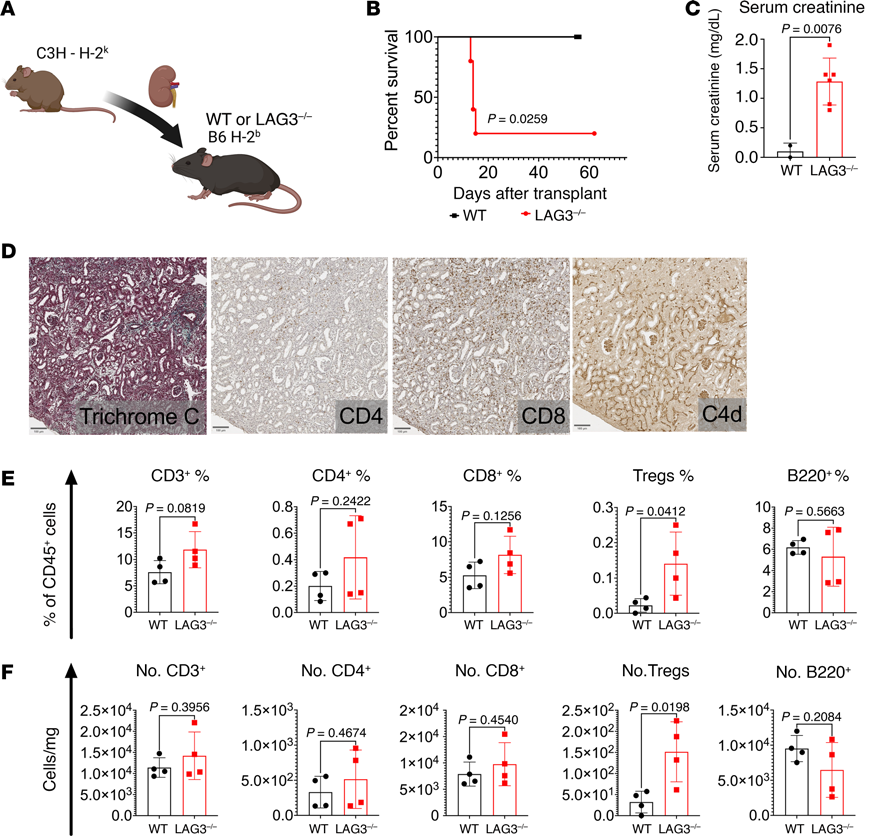
LAG3-deficient recipients acutely reject kidney allografts. Groups of B6.WT and B6.LAG3^–/–^ mice were transplanted with complete MHC-mismatched C3H kidney allografts (*n* = 4–5/group). (**A**) Kidney transplant model. (**B**) Renal allograft survival. (**C**) Serum creatinine levels at day 14 after transplant. (**D**) Renal allografts analyzed at the time of rejection (B6.LAG3^–/–^) or on day 14 after transplant (B6.WT) by trichrome C and immunoperoxidase staining for CD4, CD8, and complement component C4d. The photographs were taken at 200× original magnification (scale bars: 100 �m) and are representative of 4–5 animals in each group. (**E** and **F**) Flow cytometry analysis of graft-infiltrating immune cells: CD3^+^ (CD45^+^CD3^+^), CD4^+^ (CD45^+^CD3^+^CD4^+^), CD8^+^ (CD45^+^CD3^+^CD8^+^), Tregs (CD45^+^, CD3^+^CD4^+^FoxP3^+^), and B220^+^ (CD45^+^ B220^+^) at day 10 after transplant of C3H kidney allografts to B6.WT or B6.LAG3^–/–^ recipients. The data represent 1 of 2 experiments where each symbol represents an individual mouse. Statistical analysis of allograft survival was measured using Mantel-Cox log-rank test. For other analyses, Student’s *t* tests were performed.

To test the functional consequences of LAG3 deletion, we used a previously described mouse model of kidney transplantation in which B6 (H-2^b^) recipients undergo bilateral nephrectomy, and are then transplanted with a single fully MHC-mismatched kidney allograft (38). In this model, the recipient survival is dependent upon allograft functionality, which can be measured by serum creatinine levels. Consistent with previously published studies that used different donor strains (38, 39), B6.WT recipients spontaneously accepted C3H renal allografts for more than 60 days (Figure 2, A and B). In contrast, most B6.LAG3^–/–^ recipients rapidly rejected kidney allografts (median survival time of 15 days) (Figure 2B) and had significantly increased serum creatinine levels at day 14 (1.3 mg/dl versus 0.1 mg/dl in B6.WT controls and less than 0.4 mg/dl in nontransplanted mice, Figure 2C). Immunohistochemistry of allografts recovered from LAG3^–/–^ recipients around the time of rejection (day 14) showed decreased numbers of infiltrating T cells compared with grafts from B6.WT recipients (Supplemental Figure 3). Analysis of the graft histology showed diffuse C4d deposition in both WT and LAG3^–/–^ recipient kidney allografts (Figure 2D, Supplemental Figure 3, Supplemental Figure 4A, and Supplemental Table 1). However, LAG3^–/–^ recipient grafts had significantly increased graft edema, dilatation of the peritubular capillaries of the inner and outer cortex, and endothelial swelling (Supplemental Figure 4A and Supplemental Table 1). These findings are congruent with the Banff 2022 criteria for antibody-mediated rejection (40). Flow cytometry analysis of graft-infiltrating cells on day 10 after transplant (Figure 2, E and F) revealed no significant changes in CD4^+^ or CD8^+^ T cell infiltration, with the only significant difference being in the increased numbers of infiltrating FoxP3^+^ Tregs.

**Figure 3:**
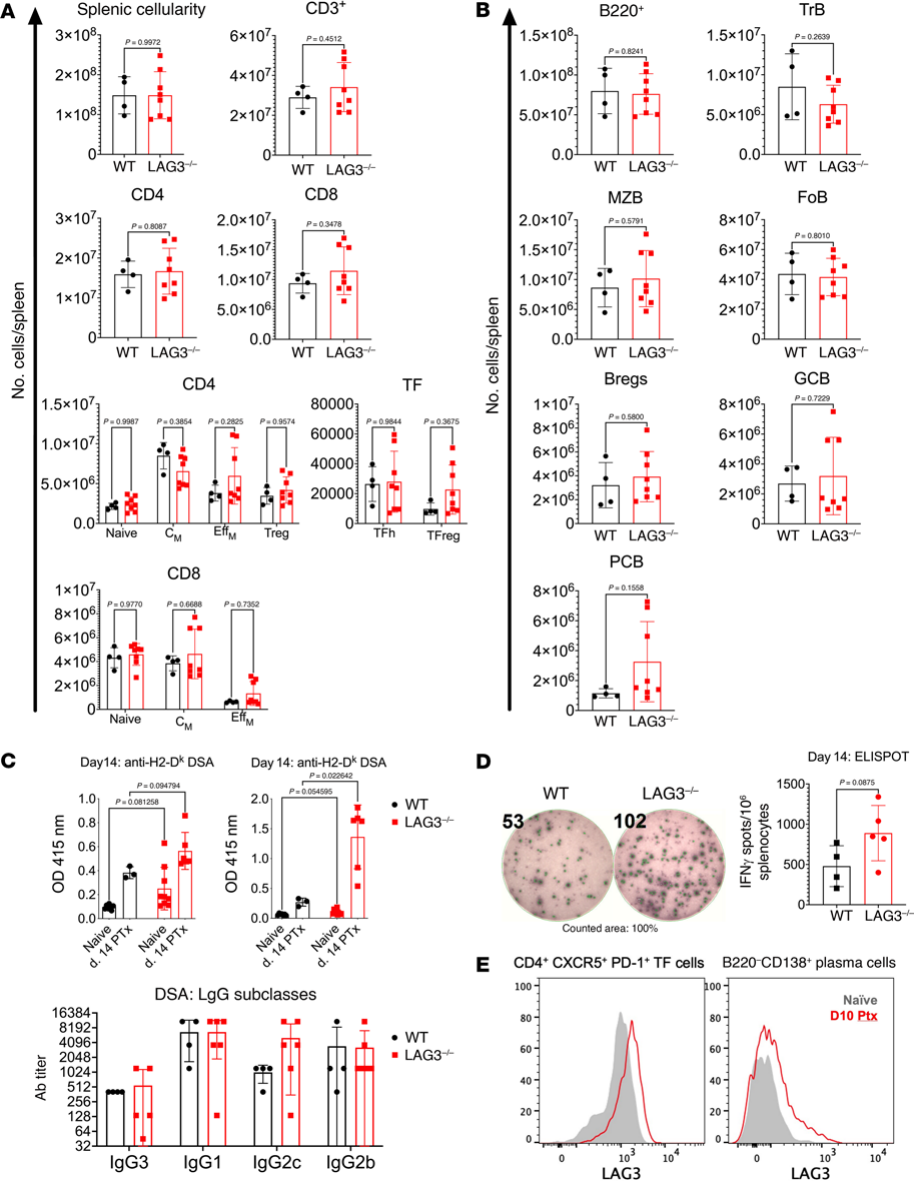
Recipient LAG3 deficiency enhances anti-donor immune responses. Analyses of donor-reactive immunity in B6.WT and B6.LAG3^–/–^ allograft recipients were performed at day 10 after transplant. (**A**) The composition of spleen T cell subsets was defined as follows: CD3 — CD3^+^, CD4 — CD3^+^CD4^+^, CD8 — CD3^+^CD8^+^, Tregs — CD3^+^CD4^+^FoxP3^+^, CD4 Naive — CD3^+^CD4^+^CD62L^hi^CD44^lo^, CD4 C_M_ — CD3^+^CD4^+^CD62L^hi^CD44^hi^, CD4 Eff_M_ — CD3^+^CD4^+^CD62L^lo^CD44^hi^, CD8 Naive — CD3^+^CD8^+^CD62L^hi^CD44^lo^, CD8 C_M_ — CD3^+^CD8^+^CD62L^hi^CD44^hi^, CD8 Eff_M_ — CD3^+^CD8^+^CD62L^lo^CD44^hi^, TFh — TCRb^+^CD4^+^FoxP3^-^PD-1^+^CXCR5^+^, and TFreg — TCRb^+^CD4^+^FoxP3^+^PD-1^+^CXCR5^+^. (**B**) The composition of splenic B cell subsets was defined as follows: B220 — B220^+^, FoB — B220^+^IgM^int^CD21/35^int^, MZB — B220^+^IgM^hi^CD21/35^hi^, TrB — B220^+^IgM^hi^CD21/35^lo^, Bregs — CD19^+^CD1d^hi^CD5^+^, GCB — B220^+^GL7^+^CD38^lo^, PCB — B220^–^ CD138^hi^. (**C**) Top: Serum levels of IgG against donor MHC-I (H2-D^k^) and MHC-II (I-A^k^). Bottom: IgG subclass analysis of serum titers of IgG3, IgG1, IgG2c, and IgG2b from WT and LAG3^–/–^ recipients at day 14 after transplant. The data are pooled from 2–3 experiments, and each symbol represents an individual mouse. (**D**) The frequencies of donor reactive IFN-γ–secreting splenocytes on day 14 after transplant. (**E**) Representative histograms of LAG3 expression by CD4^+^CXCR5^+^PD-1^+^ follicular T cells and B220^–^CD138^+^ plasma cells. Analysis of DSA responses utilized multiple unpaired *t* tests with Benjamini, Krieger, and Yekutieli false discovery approaches. For all other analyses, Student’s *t* tests were performed.

Analysis of the peripheral T cell pool revealed no significant differences in the proportion or numbers of various spleen T cell subsets (Figure 3A and Supplemental Figure 5A). Nevertheless, the absence of recipient LAG3 resulted in elevated frequencies of donor-specific T cells, as measured by IFN-γ ELISPOT assay on day 14 after transplant (Figure 3D). Analogous to the T cell compartment, spleen subsets of FoB, MZB, and TrB, and Bregs were similar in WT and LAG3^–/–^ allograft recipients (Figure 3B and Supplemental Figure 5B), whereas GCBs and plasma cells were increased in some but not all recipients. To assess anti-donor humoral immune responses, recipient serum was collected at 14 days after transplant and analyzed for the levels of IgG antibodies against donor class I (D^k^) and class II (I-A^k^) MHC molecules. Consistent with our previous research, WT renal allograft recipients had developed minimal DSAs at this time point. In contrast, LAG3^–/–^ recipients had significantly elevated levels of IgG against both D^k^ and I-A^k^ (Figure 3C). Isotype analysis showed that LAG3^–/–^ recipients had a trend toward increased levels of IFN-γ–induced T cell–dependent IgG2c, known to strongly activate FcγR and enhance antibody-dependent cellular cytotoxicity (Figure 3C) (41–44). To understand the underlying mechanisms of rejection, we analyzed LAG3 expression in recipient spleen cells on day 10 after transplant. As anticipated, effector T cells and Tregs expressed LAG3 under these conditions (data not shown and Supplemental Figure 10). Given the observed graft pathology (Figure 2D) and the serum DSA levels (Figure 3C), we measured LAG3 expression on B and T cell subsets critical to DSA generation. We found that follicular T cells and plasma cells both expressed LAG3 after transplantation (Figure 3E and Supplemental Figure 10). Taken together, these data demonstrated that LAG3 plays an essential role in regulating both cellular and humoral immune responses to transplanted allografts and contributes to spontaneous renal allograft acceptance in WT recipients.

### LAG3 regulates de novo alloresponses.

**Figure 4:**
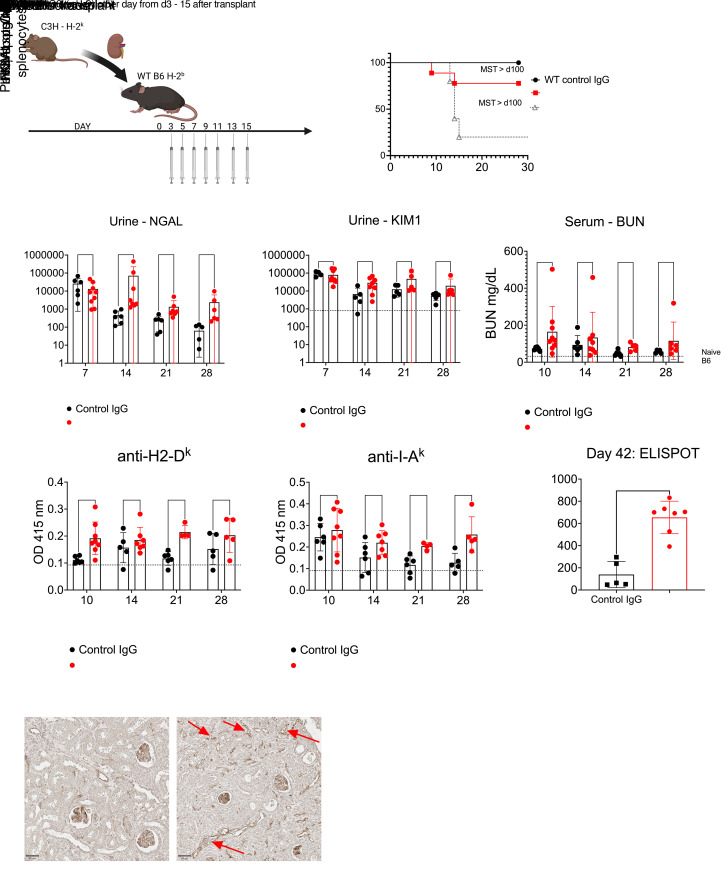
LAG3 blockade enhances de novo alloresponses after kidney transplant, leading to chronic antibody-mediated graft injury. (**A**) B6.WT mice treated with anti-LAG3 mAb (clone C9B7W) or control IgG after transplantation of C3H renal allografts. (**B**) Survival of renal allografts (*n* = 6–9/group). (**C** and **D**) NGAL and KIM1 levels in the urine collected from kidney allograft recipients. (**E**) Serum blood urea nitrogen (BUN) levels. (**F** and **G**) Serum levels of IgG against donor MHC-I (H2-D^k^) and MHC-II (I-A^k^). (**H**) The frequencies of donor-reactive IFN-γ–secreting splenocytes on day 42 after transplant. (**I**) Renal allografts harvested on day 42 after transplant and analyzed by immunoperoxidase staining for complement component C4d. Images were taken at 400× original magnification (scale bars 50 μm) and are representative of 4–5 animals in each group. The data are pooled from 2–3 experiments, and each symbol represents an individual mouse. Statistical analysis of allograft survival was measured using Mantel-Cox log-rank test. For the time-course analysis of kidney injury markers and for DSA, 1-way ANOVA with Tukey’s multiple-comparison test was performed. For ELISPOT analysis, Student’s *t* tests were performed.

Our data showed that nontransplanted LAG3-deficient mice had increased alloreactive immune responses against C3H antigens (Figure 2, A and B). To establish whether LAG3 was regulating de novo immune responses, we treated WT kidney allograft recipients with a course of LAG3 blocking antibody and monitored mice for signs of rejection (Figure 4A). LAG3 blockade resulted in rejection in 2 out 9 mice within 14 days after transplant (Figure 4B). Despite the long-term graft survival in the remaining recipients, we found that LAG3 blockade did induce graft damage, as evidenced by increased kidney injury markers NGAL and KIM1 in the urine and blood urea nitrogen (BUN) in the serum (Figure 4, C–E). Blockade of LAG3 also increased DSA production (Figure 4, F and G) and frequencies of donor-reactive T cells (Figure 4H), indicating that LAG3 regulates de novo alloresponses after transplant. The increase in alloresponses and markers of kidney injury suggested that LAG3 blockade may induce chronic injury of renal allografts. Indeed, histological analysis at day 42 after transplant (Figure 4I and Supplemental Figure 6) revealed tubular atrophy, endothelial cell swelling, and increased α-smooth muscle actin staining due to graft fibrosis, which was also reflected in the trichrome staining (Supplemental Figure 6). Although C4d staining showed C4d graft deposition in both control IgG and anti-LAG3–treated recipients, recipients that received LAG3 blockade had dilated peritubular capillaries with marginated mononuclear cells (red arrows, Figure 4I). In addition, recipients that received LAG3 blockade had increased CD4^+^ and CD8^+^ T cell graft infiltrates. These findings identified LAG3 as an important regulator of de novo immune responses to a kidney allograft.

### B cells, but not CD8^+^ T cells, are essential for kidney allograft rejection by LAG3^–/–^ recipients.

**Figure 5:**
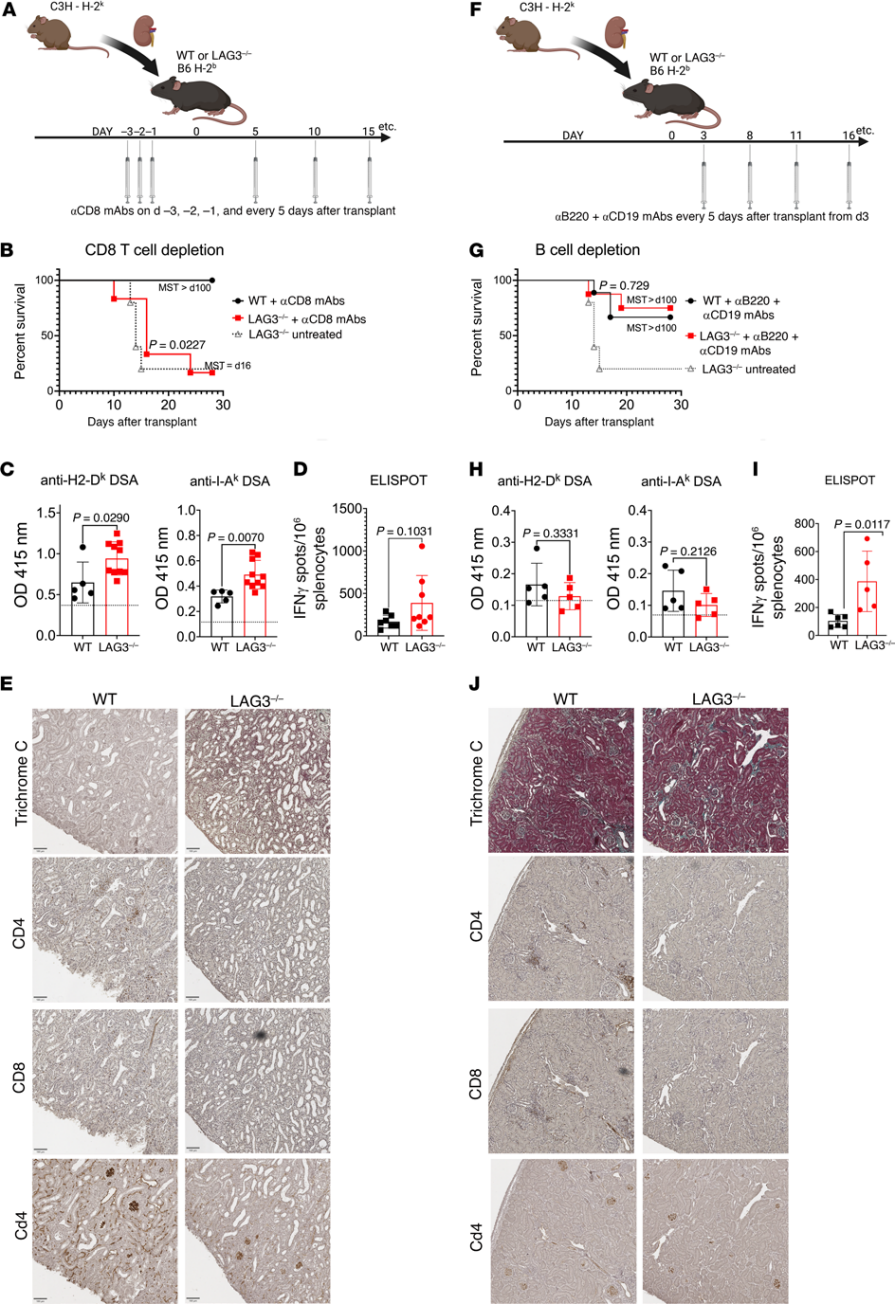
Kidney allograft rejection in LAG3-deficient recipients is dependent on B cells not CD8^+^ T cells. (**A**) B6.WT or B6.LAG3^–/–^ recipients depleted of CD8^+^ T cells prior to transplantation of C3H renal allografts. (**B**) Survival of renal allografts (*n* = 5–6/group). (**C**) Serum levels of IgG against donor MHC-I (H2-D^k^) and MHC-II (I-A^k^) in B6.WT and B6.LAG3^–/–^ kidney allograft recipients. (**D**) The frequencies of donor-reactive IFN-γ–secreting splenocytes on day 14 after transplant. (**E**) Renal allografts harvested at the time of rejection and analyzed by trichrome C and immunoperoxidase staining for CD4, CD8, and complement component C4d. Images were taken at 200× original magnification (scale bars: 100 μm) and are representative of 4–5 animals in each group. (**F**) B cells were depleted in B6.WT or B6.LAG3^–/–^ recipients after transplantation of C3H renal allografts. (**G**) Survival of renal allografts (*n* = 5–6/group). (**H**) Serum levels of IgG against donor MHC-I (H2-D^k^) and MHC-II (I-A^k^). (**I**) The frequencies of donor-reactive IFN-γ–secreting splenocytes on day 14 after transplant. (**J**) Renal allografts harvested at the time of rejection and analyzed by trichrome C and immunoperoxidase staining for CD4, CD8, and complement component C4d. Images were taken at 200× original magnification and are representative of 4–5 animals in each group. The data are pooled from 2–3 experiments, and each symbol represents an individual mouse. Statistical analysis of allograft survival was measured using Mantel-Cox log-rank test. For other analyses, Student’s *t* tests were performed.

Given that LAG3 is a well-studied regulator of T cell responses, we anticipated that LAG3 deficiency primarily affects priming of alloreactive T cells and their pathogenic functions within the kidney graft. However, the modest increase in donor-reactive T cells and the graft histology (Figures 2 and 3, and Supplemental Figure 3) suggested that alloantibodies are the major mediators of graft tissue injury in LAG3^–/–^ recipients. To formally test the contributions of cellular versus antibody-mediated rejection, LAG3^–/–^ recipients were treated with CD8^+^ T cell–depleting antibody prior to transplantation of C3H kidney allografts (Figure 5A and Supplemental Figure 7B). CD8^+^ T cell depletion antibodies failed to prolong kidney allograft survival in LAG3^–/–^ recipients, with median survival time of 16 days (Figure 5B). Anti-donor humoral immune responses were assessed at 14 days after transplant and analyzed for the levels of IgG antibodies against donor class I and class II MHC molecules. WT allograft recipients depleted of CD8^+^ T cells had a low-grade DSA response at this time point. In contrast, LAG3^–/–^ recipients had significantly elevated levels of IgG against both donor class I and class II alloantigens (Figure 5C). Although CD8^+^ T cell depletion reduced the frequencies of IFN-γ–producing T cells in both groups, LAG3-deficient recipients still had modestly elevated frequencies of donor-specific T cells on day 14 after transplant compared with WT recipients (Figure 5D). This is likely due to a faster reconstitution of LAG3^–/–^ cells following depletion, which has been previously reported in memory CD4^+^ T cells (45). Nevertheless, histological analysis at the time of rejection showed that grafts from CD8^+^ T cell–depleted LAG3^–/–^ recipients had significant damage to the kidney tubules such as tubular dilation, casts, tubular atrophy, and edema. C4d staining of these grafts showed dilated capillaries, endothelial cell swelling, and heavy damage to the tubules and to the glomeruli (Figure 5E and Supplemental Figure 8). In summary, the grafts rejected by LAG3^–/–^ recipients after CD8^+^ T cell depletion displayed the typical features of antibody-mediated graft damage similar to or even exceeding those observed in nondepleted LAG3-deficient recipients.

To test the roles of B cells and antibodies in the observed rejection, LAG3^–/–^ recipients of C3H kidney allografts were treated with anti-mouse CD19 and B220 B cell–depleting antibodies starting on day 3 after transplant and throughout the experiment (Figure 5F and Supplemental Figure 7A). Remarkably, B cell depletion restored graft survival in the majority of LAG3^–/–^ recipients (Figure 5G). Depletion of recipient B cells led to the abrogation of the DSA responses in both WT and LAG3^–/–^ recipients (Figure 5H). Although B cell depletion reduced T cell alloresponses in both groups, LAG3^–/–^ recipients still had increased frequencies of donor-reactive IFN-γ–producing T cells compared with WT recipients (Figure 5I and Figure 3D). Histological analysis at day 30 after transplant showed that recipient B cell depletion reduced allograft damage with a marked decrease in T cell infiltrates and antibody binding, demonstrated by the absence of C4d staining (Figure 5J and Supplemental Figure 9). Taken together, these results demonstrated that the rejection observed in LAG3^–/–^ mice was driven by B cell production of DSA and not by cytotoxic T cells, and that the generated DSA was a major effector mechanism of graft injury.

### LAG3 deficiency on both T and B lymphocytes is required for kidney allograft rejection.

**Figure 6:**
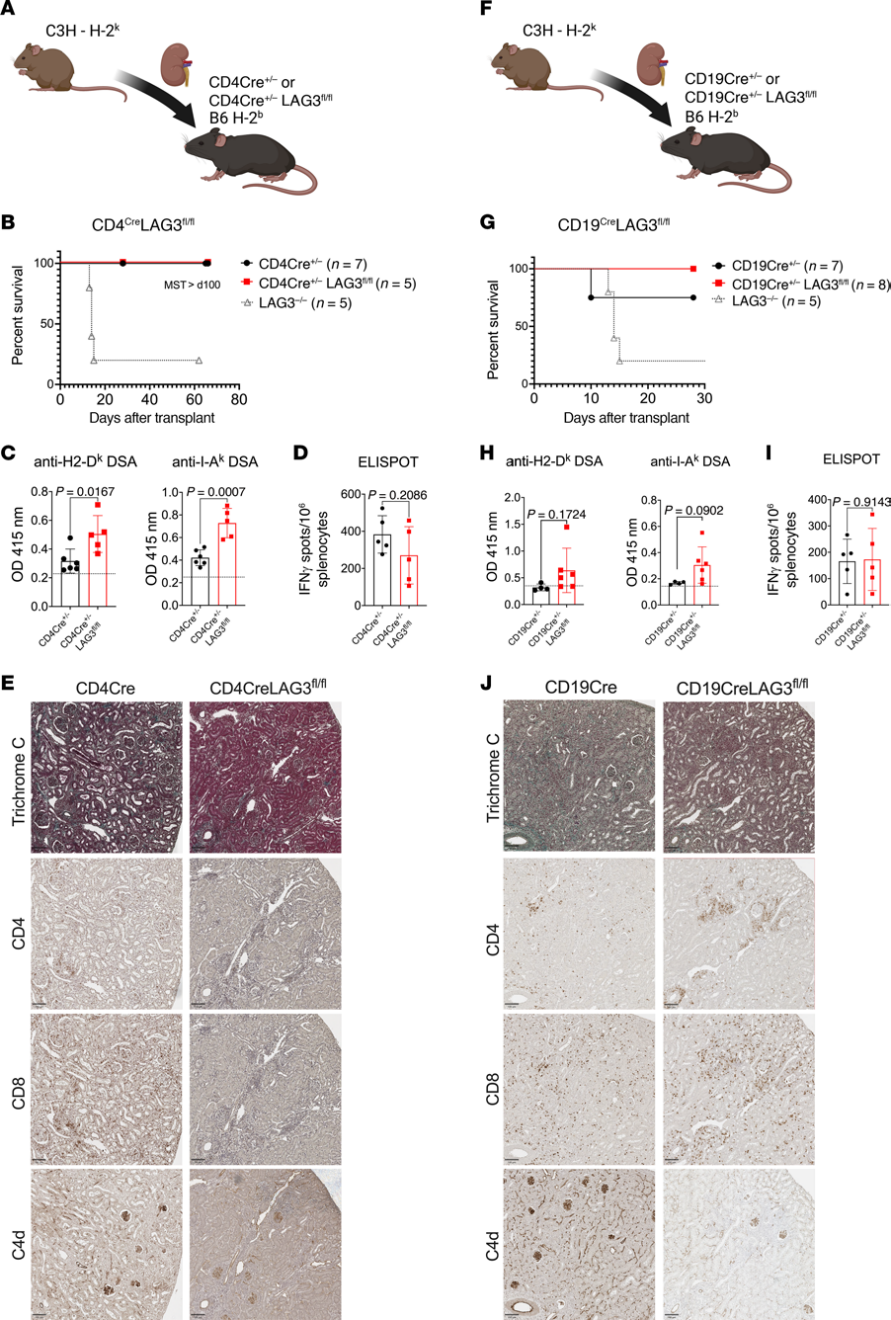
Loss of LAG3 expression on either T or B cells is not sufficient to mediate kidney allograft rejection. (**A**) B6.CD4-Cre^+/–^ or B6.CD4-Cre^+/–^LAG3^fl/fl^ recipients were transplanted with C3H renal allografts. (**B**) Survival of renal allografts (*n* = 5–7/group). (**C**) Serum levels of IgG against donor MHC-I (H2-D^k^) and MHC-II (I-A^k^) in B6.CD4-Cre^+/–^LAG3^fl/fl^ or B6.CD4-Cre^+/–^ littermate control kidney allograft recipients. (**D**) The frequencies of donor-reactive IFN-γ–secreting splenocytes on day 14 after transplant. (**E**) Renal allografts harvested at the time of rejection and analyzed by trichrome C and immunoperoxidase staining for CD4, CD8, and complement component C4d. (**F**) B6.CD19-Cre^+/–^ or B6.CD19-Cre^+/–^LAG3^fl/fl^ recipients were transplanted with C3H renal allografts. (**G**) Survival of renal allografts (*n* = 7–8/group). (**H**) Serum levels of IgG against donor MHC-I (H2-D^k^) and MHC-II (I-A^k^) in B6.CD19-Cre^+/–^ littermate controls or B6.CD19-Cre^+/–^LAG3^fl/fl^ kidney allograft recipients. (**I**) The frequencies of donor-reactive IFN-γ–secreting splenocytes on day 14 after transplant. (**J**) Renal allografts harvested at the time of rejection and analyzed by trichrome C and immunoperoxidase staining for CD4, CD8, and complement component C4d. Images were taken at 200× original magnification (scale bars: 100 μm) and are representative of 4–5 animals in each group. The data are pooled from 2–3 experiments, and each symbol represents an individual mouse. Statistical analysis of allograft survival was measured using Mantel-Cox log-rank test. For other analyses, Student’s *t* tests were performed.

The absence of LAG3 on T cells often induces pathogenesis in other mouse models (14). To address whether increased T cell help or defective Treg function was a major driver of the observed allograft injury, we used T cell conditional knockout mice and littermate controls as renal allograft recipients (Figure 6A). B6.CD4-Cre^+/–^LAG3^fl/fl^ that lack LAG3 on CD4^+^ and CD8^+^ T cells (Supplemental Figure 10B) spontaneously accepted kidney allografts for greater than 60 days (Figure 6B), indicating that LAG3 deficiency on T cells alone was not sufficient to induce rejection. Analysis of the immune response in these recipients showed elevated DSA responses (Figure 6C) but no significant changes in the frequencies of IFN-γ–producing alloreactive T cells (Figure 6D). Histological analysis of the allografts at 4 weeks after transplant revealed no major impact on fibrosis or graft tissue injury. Interestingly, despite the presence of serum DSA, T cell LAG3 deficiency resulted in reduced C4d deposition within interstitial capillaries compared with LAG3^–/–^ recipients (Figure 6E, Supplemental Figure 11, and Figure 2D).

We next investigated the role of LAG3 using a B cell conditional knockout recipient model (Figure 6F and Supplemental Figure 10). Unexpectedly, B6.CD19-Cre^+/–^LAG3^fl/fl^ recipients with specific LAG3 deficiency in B lymphocytes also failed to reject kidney allografts by day 30 after transplant (Figure 6G). LAG3 deficiency on B cells of renal transplant recipients resulted in a modest increase in DSA levels by day 14 after transplant (Figure 6H). In contrast, LAG3 deficiency on B cells had no impact on the frequencies of donor-reactive T cells at day 14 after transplant (Figure 6I), indicating that B cell LAG3 expression did not affect T cell priming. Graft histology at 4 weeks after transplant showed no significant differences in T cell infiltration. C4d staining was less intense in the CD19-Cre^+/–^LAG3^fl/fl^ recipients compared with the littermate controls but showed a rarefaction of peritubular capillaries and endothelial cell swelling consistent with antibody-mediated graft injury (Figure 6J and Supplemental Figure 12) (46). Taken together, these data showed that LAG3 expression on either helper T cells or B cells was sufficient to induce spontaneous acceptance of murine kidney allografts.

We next tested whether the loss of LAG3 on both T and B lymphocytes was sufficient to drive allograft rejection, or if another cell type was contributing to the rejection phenotype. To generate recipients in which both T and B cells lack LAG3 expression, RAG1^–/–^ mice were lethally irradiated and injected with a mixture of 9 × 10^6^ RAG1^–/–^ and 1 × 10^6^ WT or LAG3^–/–^ bone marrow cells (Supplemental Figure 13A). The reconstituted RAG1^–/–^ chimeras received C3H kidney transplants 6 weeks after adoptive transfer. Successful generation of mice in which only T and B cells lack LAG3 was confirmed by PCR (Supplemental Figure 13). As with systemic LAG3^–/–^ mice, the double conditional LAG3^–/–^ mice had increased splenic cellularity (Figure 1 and data not shown). The control chimeric mice mimicked the WT recipient survival phenotype with 100% of mice surviving to 56 days (Supplemental Figure 13A), whereas in 60% of double T and B cell conditional LAG3^–/–^ chimeric recipients, rejection occurred between day 28 and 39 after transplant. Transplant recipients with the double T and B cell LAG3 deficiency also demonstrated a trend toward increased graft damage as measured by serum BUN (Supplemental Figure 13D) and urine KIM1 (Supplemental Figure 13E). Analysis of immune responses showed no difference in serum DSA at day 21 after transplant (Supplemental Figure 13F) but a significant increase in the frequency of donor-reactive T cells from double T and B cell conditional LAG3^–/–^ chimeric recipients (Supplemental Figure 13G). Taken together, these data suggest that LAG3 expression on T and B cells is required to regulate alloimmune responses, and that the absence of LAG3 in these subsets alone is sufficient to precipitate kidney allograft rejection.

### Recipient LAG3 deficiency results in increased accumulation and effector functions of NK cells in renal allografts.

**Figure 7:**
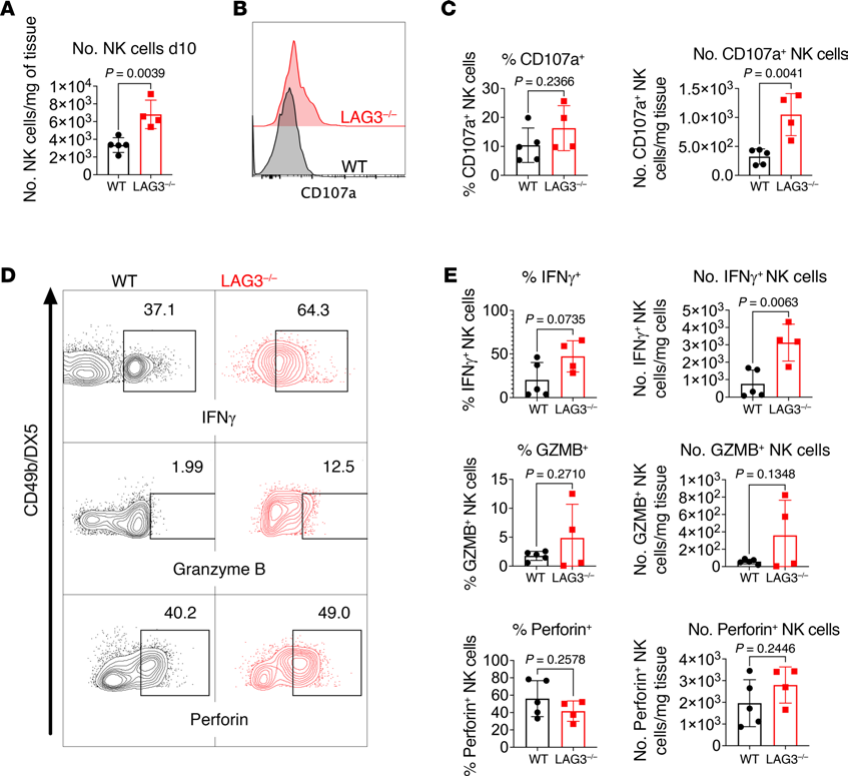
Recipient LAG3 deficiency results in increased accumulation and effector functions of NK cells in renal allografts. (**A**) Quantification of allograft-infiltrating NK cells in WT or LAG3^–/–^ recipients on day 10 after transplant. (**B**) Representative histogram and (**C**) quantification of CD107a expression by graft-infiltrating NK cells. (**D**) Representative contour plots of graft-infiltrating NK cells from WT and LAG3^–/–^ kidney allograft recipient at day 10 after transplant. (**E**) Quantification of IFN-γ, granzyme B (GZMB), and perforin producing graft-infiltrating NK cells. The gating strategy is shown in Supplemental Figure 13A. The data are pooled from 2 experiments, and each symbol represents an individual mouse. Student’s *t* tests were performed.

LAG3 was first discovered in NK cells (1), and the absence of LAG3 from NK cells may render them hyperresponsive. The critical role for NK cells during antibody-mediated tissue injury of renal allografts was recently demonstrated by Yagisawa and colleagues (47). Consistent with these findings, LAG3^–/–^ recipients had increased infiltration of NK cells, predominantly of the NK1.1^hi^ phenotype, into the graft (Figure 7A and data not shown). We next examined the effect of LAG3 deficiency on NK cell proinflammatory effector functions. LAG3^–/–^ transplant recipients had elevated numbers of NK cells expressing degranulation marker CD107a at day 10 after transplant compared with WT controls (Figure 7, B and C). We also found a significant increase in the numbers of IFN-γ–producing NK cells and a trend toward increased NK cell perforin and granzyme B production in LAG3^–/–^ versus WT recipients (Figure 7, D and E, and Supplemental Figure 14). Taken together, our findings suggest that LAG3 regulates functions of several lymphocyte subsets at different stages of antibody-mediated rejection, from DSA generation to graft tissue injury.

### LAG3 regulates antibody responses to both T cell–dependent and T cell–independent antigens.

**Figure 8:**
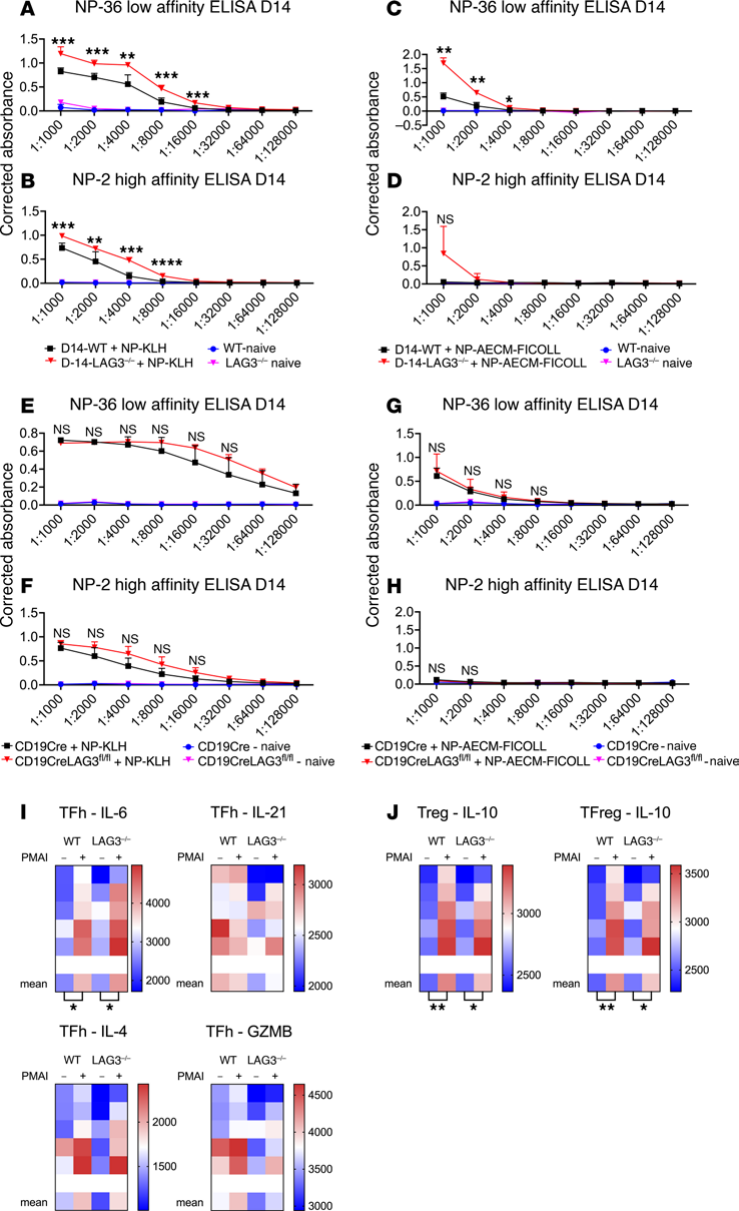
LAG3 regulates antibody responses to both T cell–dependent and T cell–independent antigens. (**A**) Serum ELISA for low-affinity NP-specific antibody was performed on sera taken at day 14 after immunization of WT and LAG3^–/–^ mice with NP-KLH. (**B**) Serum ELISA for high-affinity NP-specific antibody was performed on sera taken at day 14 after immunization of WT and LAG3^–/–^ mice with NP-KLH. (**C**) Serum ELISA for low-affinity NP-specific antibody was performed on sera taken at day 14 after immunization of WT and LAG3^–/–^ mice with NP-AECM-Ficoll. (**D**) Serum ELISA for high-affinity NP-specific antibody was performed on sera taken at day 14 after immunization of WT and LAG3^–/–^ mice with NP-AECM-Ficoll. (**E**) Serum ELISA for low-affinity NP-specific antibody was performed on sera taken at day 14 after immunization of CD19-Cre^+/–^ and CD19-Cre^+/–^LAG3^fl/fl^ mice with NP-KLH. (**F**) ELISA for high-affinity NP-specific antibody was performed on sera taken at day 14 after immunization of CD19-Cre^+/–^ and CD19-Cre^+/–^LAG3^fl/fl^ mice with NP-KLH. (**G**) ELISA for low-affinity NP-specific antibody was performed on sera taken at day 14 after immunization of CD19-Cre^+/–^ and CD19-Cre^+/–^LAG3^fl/fl^ mice with NP-AECM-Ficoll. (**H**) ELISA for high-affinity NP-specific antibody was performed on sera taken at day 14 after immunization of CD19-Cre^+/–^ and CD19-Cre^+/–^LAG3^fl/fl^ mice with NP-AECM-Ficoll. (**I**) Heatmaps of MFIs of splenic Tfh staining for IL-4, IL-21, IL-6, and granzyme B (GZMB) from WT or LAG3^–/–^ mice on day 10 after immunization with NP-KLH. (**J**) Heatmaps of MFIs of splenic Treg and Tfreg staining for IL-10 from WT or LAG3^–/–^ mice on day 10 after immunization with NP-KLH. For heatmaps, individual mice are shown above the indicated mean MFI values. Antibody dilution curves were analyzed with multiple unpaired *t* tests with Benjamini, Krieger, and Yekutieli false discovery approaches. For all other analyses, Student’s *t* tests were performed. For dilution curves, NS = not significant; in heatmaps, squares without asterisks are not significant (*P* > 0.05). *P ≤ 0.05, **P ≤ 0.01, ***P ≤ 0.001, ****P ≤ 0.0001.

To understand mechanistic aspects of LAG3 regulation of antibody responses, we next tested the impact of LAG3 deficiency on T cell–dependent versus T cell–independent antibody responses. WT or LAG3^–/–^ mice were immunized with T cell–dependent antigen 4-hydroxy-3-nitrophenylacetyl–keyhole limpet hemocyanin (NP-KLH) mixed with aluminum adjuvant or T cell–independent antigen NP-aminoethyl-carboxymethyl-Ficoll (NP-Ficoll). No difference in anti-NP IgG was found between WT and LAG3^–/–^ mice prior to immunization. In contrast, increased levels of low- and high-affinity anti-NP IgG were detected in LAG3^–/–^ mice compared with WT controls at day 14 after immunization with either T cell–dependent or T cell–independent antigen (Figure 8, A–D). LAG3^–/–^ mice had modest increases in NP-specific GCBs and memory B cells compared with WT controls 21 days after immunization with NP-KLH (Supplemental Figure 15B). No increase in splenic NP-specific plasma cells was observed at this time point (Supplemental Figure 15B). To assess the contribution of B cell LAG3 to antibody production in this model, immunization with NP-KLH or NP-Ficoll was performed in CD19-CreLAG3^fl/fl^ mice. Analogous to our findings in conditional knockout renal transplant recipients (Figure 6), CD19-CreLAG3^fl/fl^ mice had only a modest increase in anti-NP serum antibody levels after immunization with T cell–dependent (Figure 8, E and F) and T cell–independent (Figure 8, G and H) antigens relative to littermate controls, and no significant changes were observed in various NP-specific splenic B cell populations (Supplemental Figure 15C and Supplemental Figure 16C). These data support the hypothesis that LAG3-mediated regulation of antibody responses is intrinsic to both T cells and B cells. The major T cell subsets influencing antibody production are TFh, TFreg, and Treg cells. We saw no significant change in the production of IL-21, IL-4, or IL-6 by LAG3^–/–^ versus WT TFh cells at day 10 after immunization with NP-KLH (Figure 8I and Supplemental Figure 17A). Furthermore, we observed only a slight decrease in IL-10 production by both Tregs and TFregs in LAG3^–/–^ mice (Figure 8J, Supplemental Figure 17B, and Supplemental Figure 17C), suggesting changes in IL-10 production did not drive the observed increase in antibody responses. One of the mechanisms by which TFreg cells regulate antibody responses is by suppressing cytotoxic TFh cells (48). In support of this possibility, there was a trend, albeit without reaching statistical significance, toward decreased granzyme B production in the LAG3^–/–^ mice (Figure 8I and Supplemental Figure 17A).

### Plasma cell LAG3 regulates antibody production.

**Figure 9:**
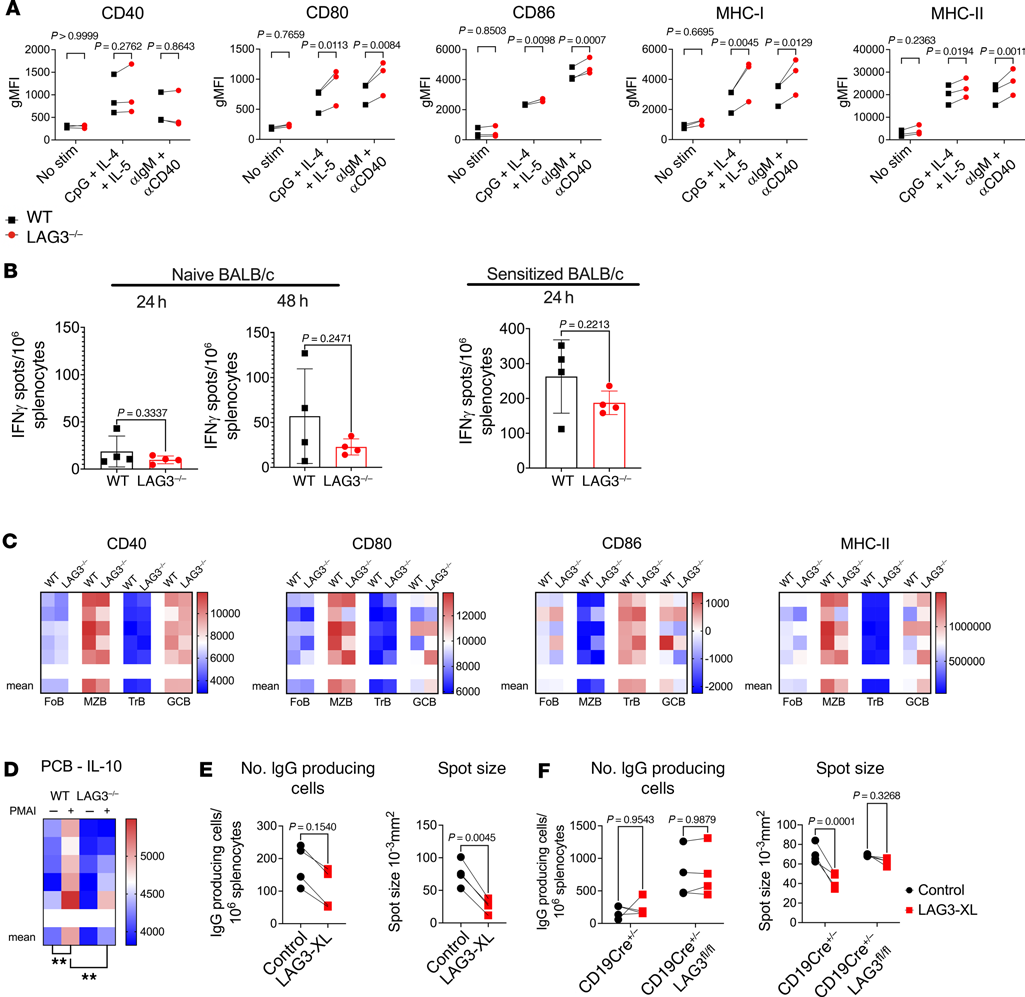
LAG3 is required for IL-10 production by plasma cells and regulates plasma cells intrinsically. (**A**) Analysis of MHC-I, MHC-II, CD40, CD80, and CD86 expression by isolated follicular B cells from WT and LAG3^–/–^ after stimulation for 24 hours with either anti-IgM and anti-CD40/CpG, IL-4, and IL-5 by flow cytometry. (**B**) ELISPOT analysis of BALB/c T cell IFN-γ responses to stimulation by either B6.WT or B6.LAG3^–/–^ isolated B cells. (**C**) Heatmaps of MFIs of CD40, CD80, CD86, and MHC-II expression by follicular B cells (FoB), marginal zone B cells (MZB), transitional B cells (TrB), and germinal center B cells (GCBs) from the spleens of WT and LAG3^–/–^ mice 10 days after immunization with NP-KLH. (**D**) Heatmaps of MFIs of IL-10 and production by splenic plasma cells (PCBs) of WT and LAG3^–/–^ mice 10 days after immunization with NP-KLH. (**E**) Quantification of frequency of IgG-producing cells and IgG spot size from plasma cell ELISPOTs of WT and LAG3^–/–^ splenocytes ± LAG3 cross-linking (LAG3-XL). (**F**) Quantification of frequency of IgG-producing cells and IgG spot size from plasma cell ELISPOTs of CD19-Cre^+/–^ and CD19-Cre^+/–^LAG3^fl/fl^ splenocytes ± LAG3 cross-linking (LAG3-XL). For heatmaps, individual mice are shown above the indicated mean MFI values. For **A**, 2-way ANOVA with Šidák’s multiple-comparison test was performed. For **B**–**E**, Student’s *t* tests were performed, and in **F**, 1-way ANOVA with Tukey’s multiple-comparison test was performed. For dilution curves, NS indicates not significant; in heatmaps, squares without asterisks are not significant (*P* > 0.05). **P ≤ 0.01.

The intrinsic role of LAG3 on B cells is poorly understood. Given that B cells can serve as APCs, we sought to address whether LAG3 regulated antigen presentation by B cells, using complementary in vitro and in vivo approaches. First, purified B cells from WT and LAG3^–/–^ mice were stimulated for 24 hours with either anti-IgM/anti-CD40 mAbs or with a CpG/IL-4/IL-5 combination (Figure 9A). In these settings, the absence of LAG3 in B cells resulted in increased expression of costimulatory molecules CD40, CD80, and CD86 as well as class I and class II MHC molecules (Figure 9A). However, we confirmed whether this affected antigen presentation by using WT or LAG3^–/–^ B cells as stimulator cells in an allogeneic T cell ELISPOT assay. LAG3^–/–^ B cells were equally as capable APCs as WT B cells, eliciting an allogenic IFN-γ response equal in magnitude (Figure 9B). Finally, we assessed the splenic B cells in vivo 10 days after immunization with NP-KLH. In contrast to in vitro stimulation experiments, there was no significant difference between WT and LAG3^–/–^ mice in the expression of CD40, CD80, CD86, and MHC-II in TrBs, MZBs, FoBs, and GCBs (Figure 9C), indicating that possible changes in B cell APC functions are not likely to account for increased humoral immune responses.

Recent studies demonstrated that LAG3^+^ plasma cells represent an IL-10–producing natural B cell subset that can regulate antibody responses (27, 49). Consistent with these reports, we detected LAG3 expression on plasma cells in renal allograft recipients (Figure 3E). We investigated whether the absence of LAG3 results in the loss of IL-10 production by plasma cells. After immunization with NP-KLH, LAG3^–/–^ mice demonstrated a significant reduction in the amount of IL-10 produced by plasma cells (Figure 9D) and a modest decrease in the frequency of IL-10^+^ splenocytes (Supplemental Figure 17D). We then tested whether LAG3 engagement affects antibody production by performing plasma cell ELISPOT measuring IgG production by preexisting plasma B cells from WT or LAG3^–/–^ mice. To signal through LAG3, cells were pretreated with an anti-LAG3 antibody and a secondary cross-linking antibody, whereas controls were treated with secondary antibody only (Figure 9, E and F). LAG3 cross-linking (LAG3-XL) in WT splenocytes reduced the frequency of detectable IgG-producing cells and the amount of IgG produced as measured by spot size (Figure 9E). To rule out the possibility that the effect was mediated through other LAG3-expressing cells, such as Tregs, we repeated the assay using splenocytes from mice with B cell–specific LAG3 deficiency. LAG3-XL had no impact on the frequency of IgG-producing cells in CD19-Cre^+/–^ control littermates (Figure 9F). In the absence of LAG3 on B cells alone, there was no effect of LAG3-XL on the frequency of IgG-producing cells. It should be noted that in the case of CD19-CreLAG3^fl/fl^, the frequency of IgG-producing cells was higher than the CD19-Cre control counterparts (Figure 9F). LAG3-XL significantly reduced the amount of IgG produced per cell by CD19-Cre controls, but had no impact on CD19-CreLAG3^fl/fl^ cells, further confirming that the effects of LAG3 engagement were plasma cell dependent (Figure 9F). These findings indicate that LAG3 can regulate B cells in an intrinsic manner and that providing agonistic signals through LAG3 can diminish antibody production by preexisting plasma cells.

## Discussion

Coinhibitory molecule LAG3 is best studied in regulating effector and regulatory T cell functions in autoimmunity, infection, and cancer (16–20, 50). However, little is known about the contribution of this pathway in regulating humoral immune responses. Our results definitively demonstrated that LAG3 regulates alloantibody generation in response to solid organ transplantation and suggest that LAG3 expression on both T and B lymphocytes plays a role in this process.

The absence of LAG3 in T cells leads to enhanced effector T cell responses and increased formation of T cell memory (26). Consistent with this, naive nontransplanted LAG3-deficient mice have elevated levels of CD44^hi^ memory T cells and enhanced response to a panel of alloantigens compared with WT counterparts (Figure 1A). In addition, we observed increased numbers of GCBs and CD138^+^ plasma cells in naive 10-week-old LAG3^–/–^ mice, which suggested ongoing B cell activation (Figure 1B). Based on previous studies, we initially proposed that LAG3 deletion in the recipient will result in dysregulated alloimmunity characterized by exaggerated activation of alloreactive T cells and leading to T cell–mediated rejection.

The model of renal transplantation was chosen for these experiments because even fully MHC-mismatched mouse kidney allografts typically do not undergo acute rejection and are spontaneously accepted in many donor/recipient strain combinations (51). Although functional C3H kidney allografts survived for more than 60 days in B6.WT recipients, they were rapidly rejected by B6.LAG3^–/–^ mice (Figure 2). Long-term survival and function of mouse renal allografts have been previously correlated with the numbers of graft-infiltrating Tregs, as FoxP3^+^ Treg depletion results in rapid graft rejection (52, 53). As anticipated, donor-reactive T cell responses were increased in the absence of LAG3 (Figure 3D), but strikingly, DSA responses were also elevated (Figure 3C). Given that we demonstrated LAG3 is expressed by TF cells and plasma B cells (Figure 3E), dysfunction in these cells could result in enhanced humoral responses.

However, as stated previously, mice with systemic deficiencies in coinhibitory receptors have an array of immune dysfunction phenotypes (54–56). Our data utilizing LAG3 blockade in kidney transplant recipients (Figure 4) supports the notion that LAG3 regulates de novo priming, and the observed dysregulation in immune responses is not due to a loss in central tolerance in the systemic knockout mice.

It is important to note that treatment of WT B6 recipients with a LAG3 blocking antibody induced a pathology consistent with chronic rejection (Figure 4). This was evident in the increased kidney injury markers in the urine and serum (Figure 4, C–E), increased serum DSA levels (Figure 4, F and G), and increased histological signs of injury (Figure 4I and Supplemental Figure 6). There is a paucity of small animal chronic injury/rejection models in kidney transplantation, with some of the current models relying on repeated administration of anti–MHC-I antibodies (57) or repeat transfers of anti-donor antibody–containing sera (58, 59). Our findings thus identified a potentially new clinically relevant model for studying the mechanisms of chronic injury and graft rejection in renal transplantation.

In contrast to germline LAG3^–/–^ mice, T cell–specific LAG3 deletion did not result in acute graft rejection (Figure 6). LAG3 is highly expressed by FoxP3^+^ Tregs, and LAG3 is thought to play a role in Tregs’ optimal suppressor activity (5, 60, 61). Previous studies demonstrated that FoxP3 Treg depletion in mouse renal allograft recipients resulted in T cell–mediated rejection without affecting DSA generation (53). Moreover, LAG3^–/–^ mice had increased numbers of FoxP3^+^ T cells before and after transplantation (Figures 1 and 2). However, it is unlikely that the augmented alloimmunity and graft rejection in our model were entirely due to dysfunctional Tregs given that specific deletion of LAG3 in recipient T cells (including Tregs) was not sufficient to induce rejection (Figure 6, A–E) (53).

Although LAG3 expression in B cells was reported in 2005 (2), its contribution to their functions has not been extensively studied. Our results demonstrated that the lack of recipient LAG3 led to increased titers of IgG DSAs and allograft rejection with characteristic features of antibody-mediated injury. Furthermore, depletion experiments confirmed that B cells were required for acute rejection of kidney allografts (Figure 5, F–J), whereas the rejection still occurred with the same kinetics in CD8^+^ T cell–depleted LAG3^–/–^ recipients (Figure 5, A–E). To date, there are only a few studies addressing the role of LAG3 in humoral immune response. Butler and colleagues (62) reported that the combination treatment with anti–PD-L1 and anti-LAG3 mAb during established malaria enhances TFh responses, plasma cell formation, and protective antibody generation in a mouse model of malaria. Our results investigating antibody production after immunizations offer key insights into the mechanisms of regulation (Figure 8 and Supplemental Figures 15–17). Notably, the absence of LAG3 systemically resulted in increased antibody responses to both T cell–dependent (Figure 8, A and B) and T cell–independent antigens (Figure 8, C and D), despite the absence of increased NP-specific plasma cells (Supplemental Figure 15B and Supplemental Figure 16B), suggesting the loss of LAG3 did not drive proliferation of antigen-specific plasma cells, but rather increased antibody production by plasma cells. This finding was corroborated by our in vitro studies showing that cross-linking of LAG3 on plasma cells diminished the amount of antibody produced by individual cells, while only modestly diminishing the frequency of antibody-secreting cells (Figure 9, E and F).

Although a potentially novel subset of LAG3^+^ IL-10–secreting plasma cells with regulatory properties has been recently reported (27), the requirement of LAG3 for IL-10 production remained to be tested. To this end, we found that IL-10 production by plasma cells was lost in immunized LAG3^–/–^ mice, suggesting LAG3 signaling on plasma cells induces the production of IL-10. Further studies are ongoing in our laboratory to determine the relative importance and the molecular mechanisms of regulation by LAG3 on Th cells versus regulatory plasma cells during the initiation of B cell activation, germinal center formation, and immunoglobulin secretion.

To understand whether LAG3 regulation of pathogenic responses leading to antibody-mediated rejection is entirely T and B cell dependent, we used a bone marrow chimera system to generate double conditional LAG3^–/–^, wherein only T and B cell lineages did not express LAG3 (Supplemental Figure 13). These recipients demonstrated no increased DSA responses (Supplemental Figure 13F), but did demonstrate increased T cell responses (Supplemental Figure 13G), likely due to a shift in immune cell composition following reconstitution (Supplemental Figure 13, H and I). Kidney transplants in these recipients were rejected, demonstrating that the absence of LAG3 on both T and B cells was sufficient to precipitate rejection.

Our study does not entirely rule out the contribution of LAG3 expressed by cells other than T and B lymphocytes. NK cells express LAG3 and are important mediators of antibody-mediated injury of renal allografts (47). Although the effects of LAG3 on various NK cell functions are highly controversial (reviewed in ref. 63), it is possible that dysregulated NK cells mediate rejection in LAG3^–/–^ recipients, secondary to enhanced T cell activation and DSA generation. Our findings support this, in that NK cells contribute to rejection as LAG3^–/–^ NK cells have increased degranulation and IFN-γ production suggestive of an increased cytotoxic state (Figure 7, B and C) and increased IFN-γ production (Figure 7, D and E). Indeed, this corroborates findings showing that the loss of LAG3 signaling in human NK cells leads to increased cytokine production (64). Taken together, these findings indicate that LAG3 on NK cells could be a target for future agonistic therapies (18) in mitigating the effector phase of the alloantibody response, which could have downstream impacts on fibrosis progression and ultimately graft outcomes.

The absence of LAG3 on recipient APCs may also contribute to elevated T cell responses. Initial analyses of LAG3 expression in DC subsets showed high LAG3 expression by plasmacytoid but not lymphoid or myeloid conventional DCs (6). However, a more recent study demonstrated a role for LAG3 on bone marrow–derived DCs in optimal T cell priming (65). Given these controversies, the impact of LAG3 deficiency in recipient APCs needs to be carefully dissected in future studies.

To our knowledge, our study is one of the first to address the role of LAG3 during alloimmune responses. Lucas et al. reported that blocking LAG3 with mAb prevents tolerization of CD8^+^ T cells after allogeneic bone marrow transplantation in mice (36). In an earlier study, the investigators used depleting anti-LAG3 mAb as an induction therapy in a rat model of cardiac transplantation. Interestingly, the treatment extended heart allograft survival, yet abrogated the tolerogenic effects of donor-specific cell transfusion, which the authors attributed to Treg depletion (37).

Despite many gaps and controversies in LAG3 biology, it is a molecule of therapeutic interest, particularly in the cancer field. Antagonistic antibodies such as relatlimab are given to patients in combination with anti–PD-1 therapy in phase II clinical trials (66). Another promising reagent undergoing phase III trials is a bispecific antibody against LAG3 and PD-1, MGD013 (28). Anti-LAG3–depleting antibodies have been developed and validated in nonhuman primates to target overactivated immune cells during undesired immune response (67). An agonistic anti-LAG3 antibody (IMP761) is currently under development for T cell–mediated autoimmunity, and this approach could have potential for clinical transplantation (18).

In conclusion, our study demonstrated the importance of LAG3 in regulating pathogenic humoral immune responses to a transplanted solid organ. The results provide a rationale for investigating LAG3 impact on B cell activation, survival, and differentiation and suggest LAG3 as a potential target for future therapeutic interventions for the prevention and treatment of antibody-mediated rejection.

## Methods

Detailed information is provided in Supplemental Methods.

### Sex as a biological variable.

Because of technical challenges in female recipients, we predominantly used male mice for our studies. However, we examined male and female animals, and similar findings were found for both sexes.

### Statistics.

Kidney allograft survival was compared between groups by Kaplan-Meier analysis. Other results were analyzed by using a parametric unpaired *t* test (2-tailed), 1-way ANOVA with Tukey’s multiple-comparison test, or multiple unpaired *t* tests with the Benjamini, Krieger, and Yekutieli false discovery approaches. The difference between groups was considered significant if the *P* value was less than 0.05. Unless noted otherwise, data are presented as mean ± SD. Total numbers of animals in each experimental group are indicated in respective figure legends.

### Study approval.

All animal studies were conducted on mice between 8 and 12 weeks of age and were approved by the IACUC of the Cleveland Clinic.

### Data availability.

All data generated in this study are available in the Supporting Data Values file.

## Author contributions

MN, JL, WMB, EC, MA, RLF, BTG, BM, and AV designed the research study. MN, RF, JL, GA, VG, JIV, YY, AB, and ND conducted the experiments. MN, JL, VG, JIV, and AB acquired the data. MN, JL, VG, JIV, and WMB analyzed the data. BM provided reagents/mice. MN, WB, BM, and AV wrote the manuscript.

## Supplementary Material

Supplemental data

Unedited blot and gel images

Supporting data values

## Version 1 05/13/2025

In-Press Preview

## Version 2 07/01/2025

Electronic publication

## References

[1] Triebel F (1990). LAG-3, a novel lymphocyte activation gene closely related to CD4. J Exp Med.

[2] Kisielow M (2005). Expression of lymphocyte activation gene 3 (LAG-3) on B cells is induced by T cells. Eur J Immunol.

[3] Durham NM (2014). Lymphocyte activation Gene 3 (LAG-3) modulates the ability of CD4 T-cells to be suppressed in vivo. PLoS One.

[4] Pena J (2014). Lymphocyte activation gene-3 expression defines a discrete subset of HIV-specific CD8+ T cells that is associated with lower viral load. AIDS Res Hum Retroviruses.

[5] Huang CT (2004). Role of LAG-3 in regulatory T cells. Immunity.

[6] Workman CJ (2009). LAG-3 regulates plasmacytoid dendritic cell homeostasis. J Immunol.

[7] Mastrangeli R (1996). Cloning of murine LAG-3 by magnetic bead bound homologous probes and PCR (gene-capture PCR). Anal Biochem.

[8] Workman CJ (2002). Phenotypic analysis of the murine CD4-related glycoprotein, CD223 (LAG-3). Eur J Immunol.

[9] Huard B (1997). Characterization of the major histocompatibility complex class II binding site on LAG-3 protein. Proc Natl Acad Sci U S A.

[10] Baixeras E (1992). Characterization of the lymphocyte activation gene 3-encoded protein. A new ligand for human leukocyte antigen class II antigens. J Exp Med.

[11] Kouo T (2015). Galectin-3 shapes antitumor immune responses by suppressing CD8+ T cells via LAG-3 and inhibiting expansion of plasmacytoid dendritic cells. Cancer Immunol Res.

[12] Xu F (2014). LSECtin expressed on melanoma cells promotes tumor progression by inhibiting antitumor T-cell responses. Cancer Res.

[13] Mao X (2016). Pathological α-synuclein transmission initiated by binding lymphocyte-activation gene 3. Science.

[14] Wang J (2019). Fibrinogen-like protein 1 is a major immune inhibitory ligand of LAG-3. Cell.

[15] Hannier S, Triebel F (1999). The MHC class II ligand lymphocyte activation gene-3 is co-distributed with CD8 and CD3-TCR molecules after their engagement by mAb or peptide-MHC class I complexes. Int Immunol.

[16] Jha V (2014). Lymphocyte Activation Gene-3 (LAG-3) negatively regulates environmentally-induced autoimmunity. PLoS One.

[17] Kim D (2019). Cutting Edge: IL-27 attenuates autoimmune neuroinflammation via regulatory T Cell/Lag3-dependent but IL-10-independent mechanisms in vivo. J Immunol.

[18] Angin M (2020). A LAG-3-specific agonist antibody for the treatment of T cell-induced autoimmune diseases. J Immunol.

[19] Grosso JF (2007). LAG-3 regulates CD8+ T cell accumulation and effector function in murine self- and tumor-tolerance systems. J Clin Invest.

[20] Goding SR (2013). Restoring immune function of tumor-specific CD4+ T cells during recurrence of melanoma. J Immunol.

[21] Guy C (2022). LAG3 associates with TCR-CD3 complexes and suppresses signaling by driving co-receptor-Lck dissociation. Nat Immunol.

[22] Ming Q (2022). LAG3 ectodomain structure reveals functional interfaces for ligand and antibody recognition. Nat Immunol.

[23] Adam K (2024). Cutting Edge: LAG3 dimerization is required for TCR/CD3 interaction and inhibition of antitumor immunity. J Immunol.

[24] Gagliani N (2013). Coexpression of CD49b and LAG-3 identifies human and mouse T regulatory type 1 cells. Nat Med.

[25] Liang B (2008). Regulatory T cells inhibit dendritic cells by lymphocyte activation gene-3 engagement of MHC class II. J Immunol.

[26] Zhang Q (2017). LAG3 limits regulatory T cell proliferation and function in autoimmune diabetes. Sci Immunol.

[27] Lino AC (2018). LAG-3 inhibitory receptor expression identifies immunosuppressive natural regulatory plasma cells. Immunity.

[28] LaMotte-Mohs R (2016). MGD013, a bispecific PD-1 x LAG-3 Dual-Affinity Re-Targeting (DART®) protein with T-cell immunomodulatory activity for cancer treatment. Cancer Res.

[29] Savitsky D (2018). Abstract 3819: INCAGN02385 is an antagonist antibody targeting the co-inhibitory receptor LAG-3 for the treatment of human malignancies. Cancer Res.

[30] Zettl M (2018). Abstract 4547: Characterization of the LAG-3 targeting antibody BI 754111 in monotherapy and in combination with the anti-PD-1 antibody BI 754091. Cancer Res.

[31] Hong DS (2018). Phase I/II study of LAG525 ± spartalizumab (PDR001) in patients (pts) with advanced malignancies. J Clin Oncol.

[32] Kraman M (2020). FS118, a bispecific antibody targeting LAG-3 and PD-L1, enhances T-cell activation resulting in potent antitumor activity. Clin Cancer Res.

[33] Brignone C (2010). First-line chemoimmunotherapy in metastatic breast carcinoma: combination of paclitaxel and IMP321 (LAG-3Ig) enhances immune responses and antitumor activity. J Transl Med.

[34] Legat A (2016). Vaccination with LAG-3Ig (IMP321) and peptides induces specific CD4 and CD8 T-cell responses in metastatic melanoma patients — report of a phase I/IIa clinical trial. Clin Cancer Res.

[35] Romano E (2014). MART-1 peptide vaccination plus IMP321 (LAG-3Ig fusion protein) in patients receiving autologous PBMCs after lymphodepletion: results of a Phase I trial. J Transl Med.

[36] Lucas CL (2011). LAG-3, TGF-β, and cell-intrinsic PD-1 inhibitory pathways contribute to CD8 but not CD4 T-cell tolerance induced by allogeneic BMT with anti-CD40L. Blood.

[37] Haudebourg T (2007). Depletion of LAG-3 positive cells in cardiac allograft reveals their role in rejection and tolerance. Transplantation.

[38] Gorbacheva V (2016). Memory CD4 T cells induce antibody-mediated rejection of renal allografts. J Am Soc Nephrol.

[39] Bickerstaff A (2008). Acute humoral rejection of renal allografts in CCR5(-/-) recipients. Am J Transplant.

[40] Cendales LC (2024). Banff 2022 vascularized composite allotransplantation meeting report: diagnostic criteria for vascular changes. Am J Transplant.

[41] Collins AM (2016). IgG subclass co-expression brings harmony to the quartet model of murine IgG function. Immunol Cell Biol.

[42] Klaus GG (1979). Activation of mouse complement by different classes of mouse antibody. Immunology.

[43] Slifka MK, Ahmed R (1996). Long-term antibody production is sustained by antibody-secreting cells in the bone marrow following acute viral infection. Ann N Y Acad Sci.

[44] Murata K (2007). Synergistic deposition of C4d by complement-activating and non-activating antibodies in cardiac transplants. Am J Transplant.

[45] Previte DM (2019). Lymphocyte Activation Gene-3 maintains mitochondrial and metabolic quiescence in naive CD4^+^ T Cells. Cell Rep.

[46] Li X (2014). Capillary dilation and rarefaction are correlated with intracapillary inflammation in antibody-mediated rejection. J Immunol Res.

[47] Yagisawa T (2019). In the absence of natural killer cell activation donor-specific antibody mediates chronic, but not acute, kidney allograft rejection. Kidney Int.

[48] Xie MM (2019). Follicular regulatory T cells inhibit the development of granzyme B-expressing follicular helper T cells. JCI Insight.

[49] Manakkat Vijay GK (2024). Temporal dynamics and genomic programming of plasma cell fates. Nat Immunol.

[50] Maruhashi T (2020). LAG-3: from molecular functions to clinical applications. J Immunother Cancer.

[51] Tse GH (2013). Systematic review of mouse kidney transplantation. Transpl Int.

[52] Alessandrini A, Turka LA (2017). FOXP3-positive regulatory T cells and kidney allograft tolerance. Am J Kidney Dis.

[53] Miyajima M (2011). Early acceptance of renal allografts in mice is dependent on foxp3(+) cells. Am J Pathol.

[54] Chambers CA (1997). Lymphoproliferation in CTLA-4-deficient mice is mediated by costimulation-dependent activation of CD4+ T cells. Immunity.

[55] Nishimura H (1998). Immunological studies on PD-1 deficient mice: implication of PD-1 as a negative regulator for B cell responses. Int Immunol.

[56] Joller N (2011). Cutting edge: TIGIT has T cell-intrinsic inhibitory functions. J Immunol.

[57] Fukami N (2009). Antibodies to MHC class I induce autoimmunity: role in the pathogenesis of chronic rejection. J Immunol.

[58] Akiyoshi T (2012). Role of complement and NK cells in antibody mediated rejection. Hum Immunol.

[59] Chong AS (2013). Lessons and limits of mouse models. Cold Spring Harb Perspect Med.

[60] Workman CJ, Vignali DA (2005). Negative regulation of T cell homeostasis by lymphocyte activation gene-3 (CD223). J Immunol.

[61] Camisaschi C (2010). LAG-3 expression defines a subset of CD4(+)CD25(high)Foxp3(+) regulatory T cells that are expanded at tumor sites. J Immunol.

[62] Butler NS (2011). Therapeutic blockade of PD-L1 and LAG-3 rapidly clears established blood-stage Plasmodium infection. Nat Immunol.

[63] Khan M (2020). NK cell-based immune checkpoint inhibition. Front Immunol.

[65] Garcia Cruz D (2021). Lymphocyte activation Gene-3 regulates dendritic cell metabolic programing and T cell priming function. J Immunol.

[66] Tawbi HA (2022). Relatlimab and nivolumab versus nivolumab in untreated advanced melanoma. N Engl J Med.

[67] Poirier N (2011). Antibody-mediated depletion of lymphocyte-activation gene-3 (LAG-(3+))-activated T lymphocytes prevents delayed-type hypersensitivity in non-human primates. Clin Exp Immunol.

