# Supplementary material for: LAG3 regulates antibody responses in a murine model of kidney transplantation
Source: J Clin Invest. 2025 May 13;135(13):e172988. doi: 10.1172/JCI172988 (PMC12208549; doi:10.1172/JCI172988)
Supplement: Supplemental data [file jci-135-172988-s162.pdf]

Extended Methods

Animals

C3H/HeJ (C3H, H-2^k), C57BL/6J (B6, H-2^b), BALB/cJ (Balb/c, H-2^d) DBA1/J (DBA H-2^q) and SJL/J-*Pde6b*^{rd1} (SJL, H-2^s), B6.129P2(C)-Cd19 tm1(cre)Cgn/J (CD19Cre), B6.RAG1$^{-/-}$, B6.LAG3$^{-/-}$ aged 4-8 week, were purchased from The Jackson Laboratory (Bar Harbor,ME). LAG3$^{fl/fl}$ mice were commissioned by Biocytogen (Beijing, China) and bred to CD4Cre or CD19Cre mice to generate T cell or B cell conditional LAG3$^{-/-}$ mice respectively. All the mouse protocols were approved by the Institutional Animal Care and Use Committee (IACUC) of the Cleveland Clinic. The mice were maintained under specific pathogen-free conditions at the Cleveland Clinic Lerner Research Institute Biological Resource Institute.

Kidney Transplantation and Graft Evaluation

Transplantation of murine kidneys was performed as previously described [1]. In brief, the kidney and its vascular supply and ureter were harvested *en bloc*, the donor vasculature were anastomosed to the recipient abdominal aorta and inferior vena cava. Excised kidneys were perfused with University of Wisconsin (UW) solution (320mOsm; Preservation Solutions, Elkorn, WI) and stored on ice for 0.5 hours before transplantation. Urinary reconstruction was performed as previously published [2]. The remaining native kidney was removed at the time of transplant, ensuring that survival of the mouse was dependent upon functionality of the transplanted kidney. Graft survival was assessed by daily examination of overall animal health. Recipients were euthanized when low and slow mobility in addition to a hunched posture indicated rejection. At time of euthanasia serum creatinine levels were measured using the VetScan i-STAT1 Analyzer (Abaxis, Union City, CA). Grafts were collected and fixed in methanol before embedding in paraffin for staining with hematoxylin and eosin or Gomori's trichrome C, and the following

antibodies: rabbit monoclonal anti-mouse/human Mac-2 (clone M3/M8; Biolegend #125401), rabbit monoclonal anti-mouse CD4 (clone EPR19514; Abcam #ab183685), rabbit monoclonal anti-mouse CD8 (clone EPR21769; Abcam #ab217344), polyclonal rabbit anti-mouse C4d [3], rabbit polyclonal anti-Human/mouse vWf (Dako #A0082), rat monoclonal anti-mouse/rat FOXP3 (clone FJK-16s; eBioscience #14-5773) and rat monoclonal anti-mouse B220/CD45R (clone RA3-682; BD Pharmingen #550286). Histology slides were scanned using a Leica Biosystems Aperio AT2 slide scanner, and images were selected and cropped using QuPath slide analysis software. Each stain was evaluated based on two to three complete cross sections (whole slides) from 4-5 grafts per group, and the scoring was performed as outlined in Supplementary Table 1 (**Table S1**) by a pathologist (WMB) without previous knowledge of experimental groups. Blood urea nitrogen (BUN) levels were measured using BUN Colorimetric Detection Kit (Thermo Fisher Scientific, Waltham, MA). Urine levels of TIM-1/KIM-1/HAVCR and Lipocalin-2/NGAL were measured with Quantikine ELISA kits (R&D System Inc., Minneapolis, MN) as previously published [4].

Recipient Treatment

For LAG3 blockade, anti-LAG-3 mAb (clone C9B7w, Bio X Cell) [5,6] was given i.p. at a dose of 100 µg on days 3, 5, 7, 9, 11, 13 and 15 posttransplant. To deplete $CD8^+$ T cells recipients were treated with monoclonal anti-mouse CD8 antibodies, clones TIB105 and YTS169 (Bio X Cell, 0.2mg of each i.p. on days -3, -2, -1 prior to transplantation and every 5 days posttransplant for the duration of the experiment). For B cell depletion, recipients were treated with anti-mouse CD19 clone 1D3 and anti-mouse B220 clone RA3.3A1/6.1 (TIB-146) monoclonal antibodies Bio X Cell, 0.2mg of each i.p. on day 3 and every 5 days posttransplant for the duration of the experiment).

Measurement of Serum Alloantibody

Alloreactive serum antibody titers were measures as previously published [7]. In brief, serum samples were collected via tail-vein bleeding from naïve 10 weeks old mice or from kidney allograft recipients at d. 14 posttransplant and stored at -20°C. Flat-bottomed 96-well plates (Thermo Fisher Scientific) were coated overnight at 4°C with biotinylated class 1 D^d (folded with RPGGRAFVTI peptide), class 1 D^k (folded with RRLGRTLLL peptide), class 2 I-A^d (folded with PVSKMRMATPLLMQA class 2-associated invariant chain peptide), or class 2 I-A^k (folded with PVSKMRMATPLLMQA class 2- associated invariant chain peptide) MHC monomers at 1μg/ml in PBS. Peptide/MHC monomers were provided by the National Institutes of Health Tetramer Core Facility at Emory University (Atlanta, GA). Plates were washed with PBS and blockade with 1% BSA-PBS solution for 1h at room temperature. A total of 100μl of serum diluted from 1:100-1:500 were added to the plates and incubated overnight at 4°C. Plates were washed with PBS and PBS-Tween (0.25%), and then incubated with goat anti-mouse IgG -horse radish peroxidase (HRP) conjugate (1:20000 dilution; Thermo Fisher Scientific) or IgG1, IgG2a, IgG2b, IgG3-horse radish peroxidase (HRP) conjugate (1:5000 dilution; Thermo Fisher Scientific) for 1 hour at room temperature. After incubation with conjugate-bound antibodies, plates were washed and developed with ABTS Peroxidase Substrate (KPL, Gaithersburgh, MD). Absorbance was measured at 415nm using the Bio-Rad iMark Microplate Reader (Bio-Rad Laboratories, Hercules, CA). For IgG isotypic analysis, donor C3H, third party SJL and congenic B6.K^d thymocytes were isolated and 1x106 cell aliquots were incubated with 100µl of serially diluted recipient serum or with non-diluted cell culture supernatant. Anti-mouse IgG1, IgG2c, IgG3 and IgG2b were used at a 1:50 – 1:100 dilution (BD Pharmingen). Cells were washed, fixed in 1% paraformaldehyde, and analyzed by flow cytometry. For every sample and every IgG isotype, the mean fluorescence intensity (MFI) of each dilution was determined. The dilution that returned the MFI to the level

observed when thymocytes were stained with a 1:90 dilution of naïve WT B6 serum was divided by two and reported as the titer.

ELISPOT Assay

Interferon gamma (IFNγ) ELISPOT assays were performed as previously described using capture and detecting antibody from BD Pharmingen [8]. Spleens from recipients or naïve non-transplanted mice were stimulated with mitomycin C-treated donor C3H or BALB/c, SJL or DBA spleen cells for 24 hours. The resulting spots were analyzed using an ImmunoSpot Series 4 Analyzer (Cellular Technology, Cleveland, OH). The frequencies of IgG antibody secreting cells (ASC) were determined using ELISpot PLUS for mouse IgG and IgM kits (MABTECH AB, Nacka Strand, Sweden) as previously published [9,10]. In brief, splenocytes were isolated from WT, CD19Cre$^{+/-}$, and CD19CreLAG3$^{fl/fl}$ mice and, were incubated on ice with either media or rat anti-LAG-3 mAb (clone C9B7w, Bio X Cell) [6] at 5μg/ml for 90 minutes on ice. Cells were then washed and incubated with rabbit anti-rat IgG (Invitrogen, Waltham, MA) at 25μg/ml for 90 minutes on ice, and then cultured for 24 h in RPMI media supplemented with 5% of Fetal Bovine Serum (FBS) in 96-well plates coated with anti-mouse IgG Ab. Biotinylated-anti-IgG was added as detection reagent for two hours at room temperature. After extensive washes, the plates were incubated with Streptavidin-Alkaline Phosphatase for one hour at room temperature followed by BCIP/NBT substrate. The numbers of spots per well and the cumulative spot size distribution were analyzed using an ImmunoSpot Series 4 Analyzer (Cellular Technology Ltd., Cleveland, OH).

Flow Cytometry

Fluorochrome conjugated antibodies were purchased from BD Biosciences (San Jose, CA) or eBioscience (San Diego, CA). Cells were isolated from the spleen and stained as previously described [11], ≥100,000 events per sample were acquired on a BD Biosciences LSRII or a BD

Biosciences LSRFortessa X-20 followed by data analysis using FlowJo software (TreeStar, Inc., Ashland, OR).

Graft Infiltrating Cell Analysis

Cells were isolated from grafts on day 10 posttransplant as previously described [12-14], counted and plated in 96 well plates in either complete RPMI or in complete RPMI containing eBioscience Cell Stimulation Cocktail plus Protein Transport Inhibitors (Thermofisher #00-4975-93) for 4 hours at 37°C. Cells were then washed and stained for flow cytometry analysis.

Bone Marrow Chimera Generation

Bone marrow was isolated from femurs of wild type B6, B6.LAG3$^{-/-}$ and B6.RAG1$^{-/-}$ mice. RAG1$^{-/-}$ mice were irradiated (1100rads) and injected i.v. with bone marrow cells so that each recipient received 9 x 10^6 RAG1$^{-/-}$ cells plus 1 x 10^6 either WT or LAG3$^{-/-}$ cells as previously described [12]. 6 weeks later, the reconstituted chimeras were transplanted with C3H kidney allografts. Chimerism was confirmed on flow sorted cells by PCR.

In vitro B cell stimulations

Splenocytes from 8-10-week-old mice were obtained by mechanical disruption of the spleen between 2 frosted glass slides in FACS buffer (PBS, 0.1% BSA, 1 mM EDTA) followed by filtration through 0.66 μm nytex mesh and lysed in ACK buffer. Follicular B cells were positively selected with anti-CD23-biotin mAb (Biolegend, clone: B3B4, #101604) and MACS column selection. Enriched follicular B cells were cultured in complete growth media (RPMI, 10%FBS, HEPES, Penn/Strep, MEM non-essential amino acids, L-glutamine, Na-Pyruvate, 2-ME). Cells were plated at 1 × 10^6 cells/mL in 96-well culture plates at 200 μL/well. Cultures were supplemented as indicated with CpG DNA (0.1 μM), IL-4 (100 ng/mL), and IL-5 (100 ng/mL), or anti IgM (10μg/ml,

AffiniPure^{TM} F(ab')$_2$ Fragment Goat anti-mouse IgM, m chain specific, Jackson ImmunoResearch, #115-006-020) and anti-CD40(10μg/ml, InVivoPlus anti-mouse CD40, BioXcell, clone: FGK4.5, #BP0016-2) Abs . Cultures were incubated at 37°C, 5% CO_2 for 24 hours. Cells were then stained for flow cytometry as described for other experiments. Each data point is the mean of three technical replicates, each point represents an individual mouse.

Immunizations

Wild type B6, B6.LAG3$^{-/-}$, B6.CD19Cre$^{+/-}$ or B6.CD19Cre$^{+/-}$LAG3$^{fl/fl}$ mice were immunized i.p. with 50μg of either 4-Hydroxy-3-nitrophenylacetyl (NP)-Keyhole Limpet Hemocyanin (NP-KLH) in alum or NP-AminoEthylCarboxtyMethyl-FICOLL (NP-FICOLL) (Biosearch Technologies). Serum was taken from tail vein on day 0 (unimmunized), 7, 14 and 21 after immunization. NP-specific antibody titers we measured by ELISA. In brief, capture antigen (NP2-BSA or NP36-BSA, Biosearch Technologies) was coated to flat-bottomed NuncTM plates (Thermofisher Scientific) overnight at 4°C. After washing and blocking, serum was plated at various dilutions for 1 hour at room temperature. Plates were then washed and then incubated with detection antibody (Southern Biotech – Goat Anti-Mouse IgG, Human adsorbed – Biotinylated - #1030-08) at room temperature for 2 hours. Plates were then washed and before the addition of HRP-streptavidin (Thermofisher #N100) for 1hr at RT, after a wash, wells were developed with the addition of 100/μl well of TMB substrate (BD Biosciences – BD OptEIA TMB Substrate Reagent kit #555214) solution, the reaction was stopped with the addition of 1M phosphoric acid. The data was analyzed using the Agilent Biotek Epoch plate reader, reading the absorbance at 450nm subtracting the background at 550nm. Immunized mice were sacrificed on day 21 after immunization and immune cells were enumerated by flow cytometry, and cytokine production by cells following immunization was performed in line with other described experiments.

Table S1.

Histological Indication	Score	Description
Edema	0	No edema
	1	Some edema in the interstitium
	2	Edema in 25-50% of the interstitial space
	3	Edema in the majority of the interstitum
C4d Diffuseness	0	Insignificant C4d staining
	1	Minimal C4d staining 1<10% of staining area
	2	Focal C4d staining 10-50% of staining area
	3	Diffuse >50% of staining area
Dilatation of peritubular capillaries (PTC)	0	Little or no capillary changes
	1	Limited dilatation of peritubular capillaries
	2	Dilatation in 25-50% peritubular capillaries
	3	Dilatation in the majority of peritubular capillaries
PTC Endothelial cell swelling	0	No observed endothelial swelling
	1	Some PTC endothelial cell swelling
	2	Swelling in 25-50% of observed PTC endothelial cells
	3	Swelling in the majority of PTC endothelial cells

Supplementary Figures

Figure S1.

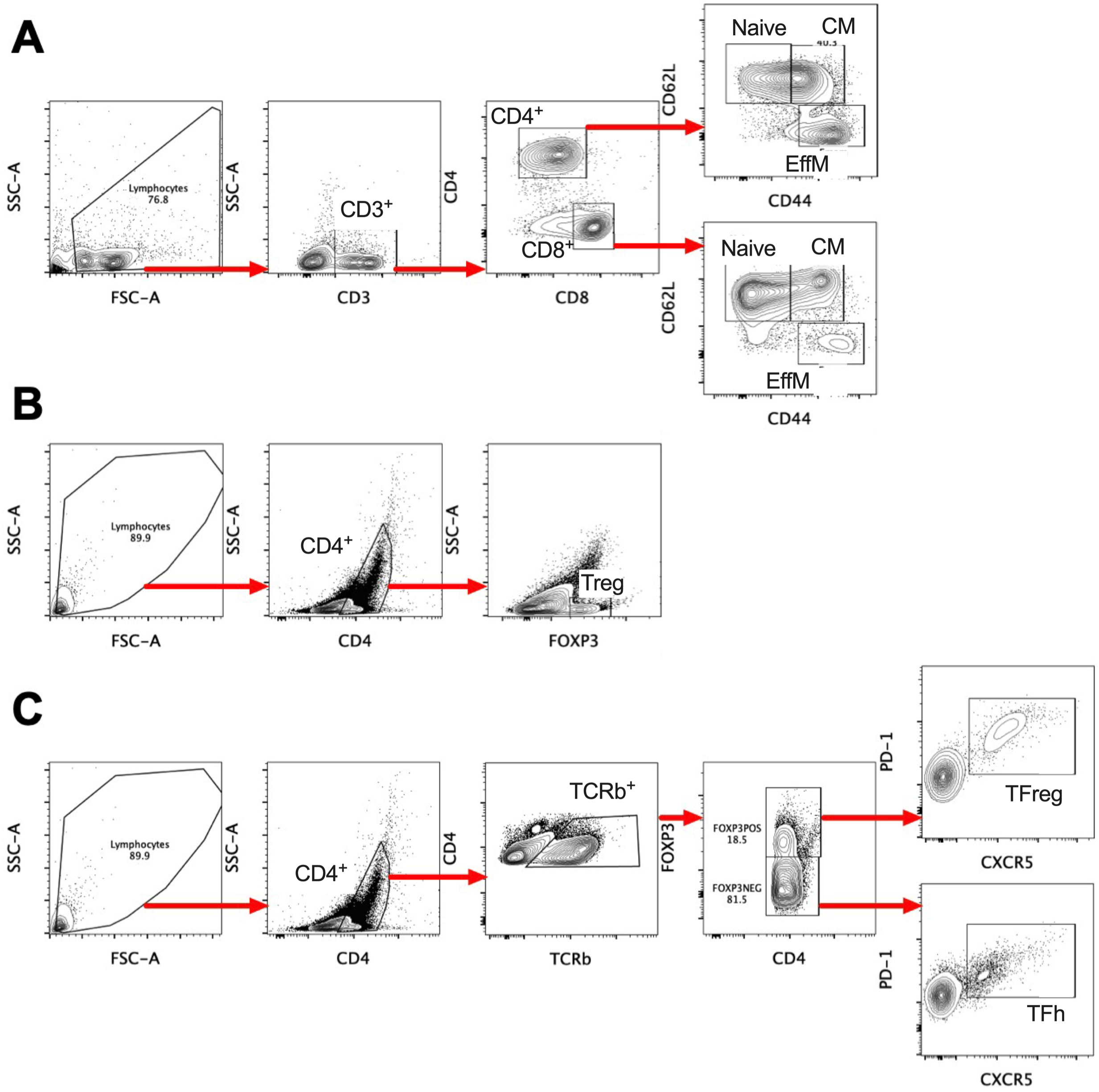

Figure S1. Splenic T cell gating strategies. ***A***. *CD4 (CD3+CD4+), CD8 (CD3+CD8+), CD4 TEffM (CD3+, CD4+, CD62Llo, CD44hi), CD8 TEffM (CD3+, CD8+, CD62Llo, CD44hi).* ***B***. *Tregs (CD3+, CD4+, FoxP3+).* ***C***. *TFh (TCRb+, CD4+, FoxP3-, PD-1+, CXCR5+) and TFreg cells (TCRb+, CD4+, FoxP3+, PD-1+, CXCR5+).*

Figure S2.

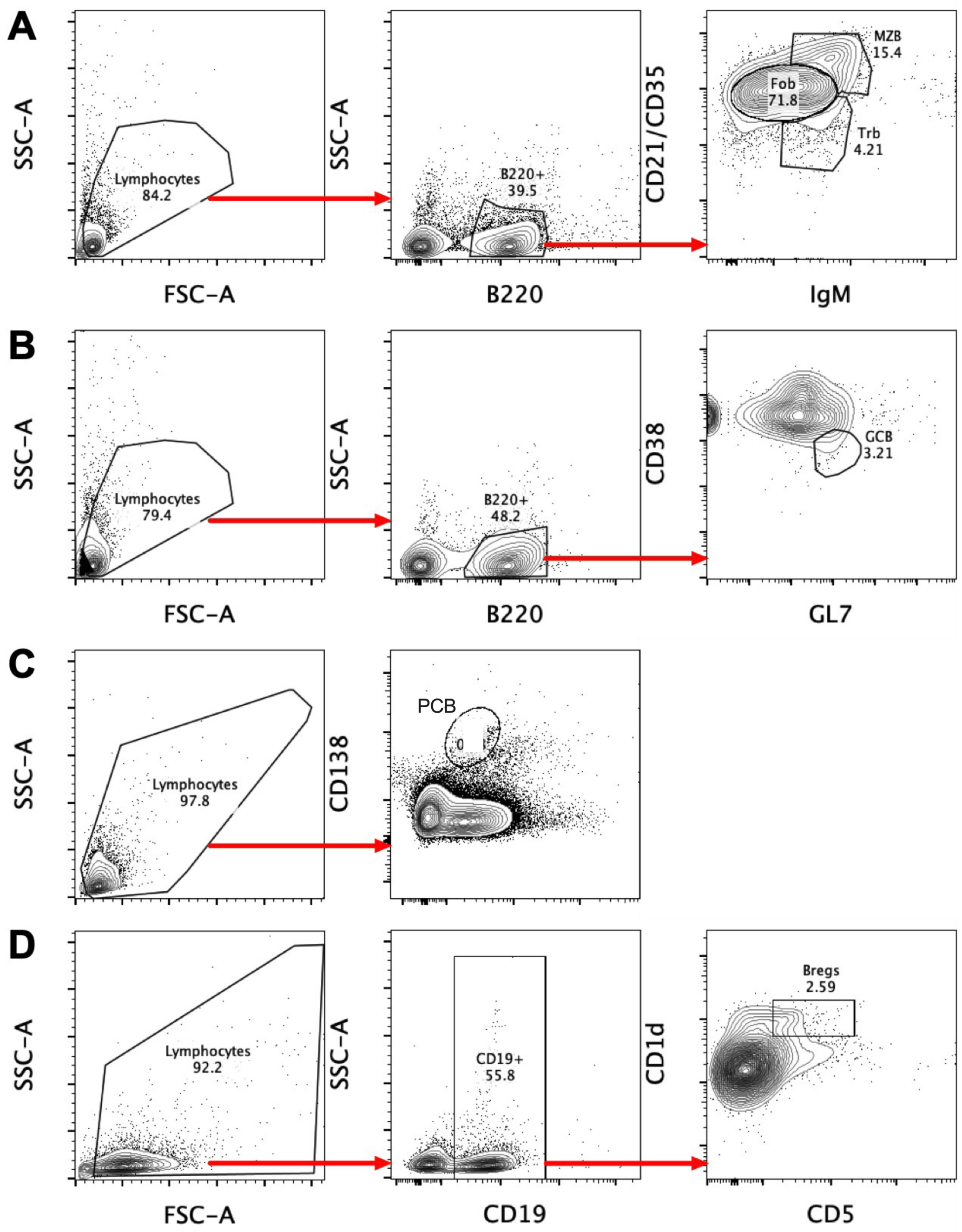

Figure S2. Splenic B cell gating strategies. *Subsets were defined as follows;* ***A****. FoB - B220*$^{+}$*, IgM*^{int}*, CD21/35*^{int}*, MZB - B220*$^{+}$*, IgM*^{hi}*, CD21/35*^{hi}*,TrB - B220*$^{+}$*, IgM*^{hi}*, CD21/35*^{lo}*.* ***B****. GCB - B220*$^{+}$*, GL7*$^{+}$*, CD38*^{lo}*.* ***C****. PCB –B220*$^{-}$*, CD138*^{hi}*.* ***D****. Bregs - CD19*$^{+}$*,CD1d*^{hi}*, CD5*$^{+}$*.*

Figure S3

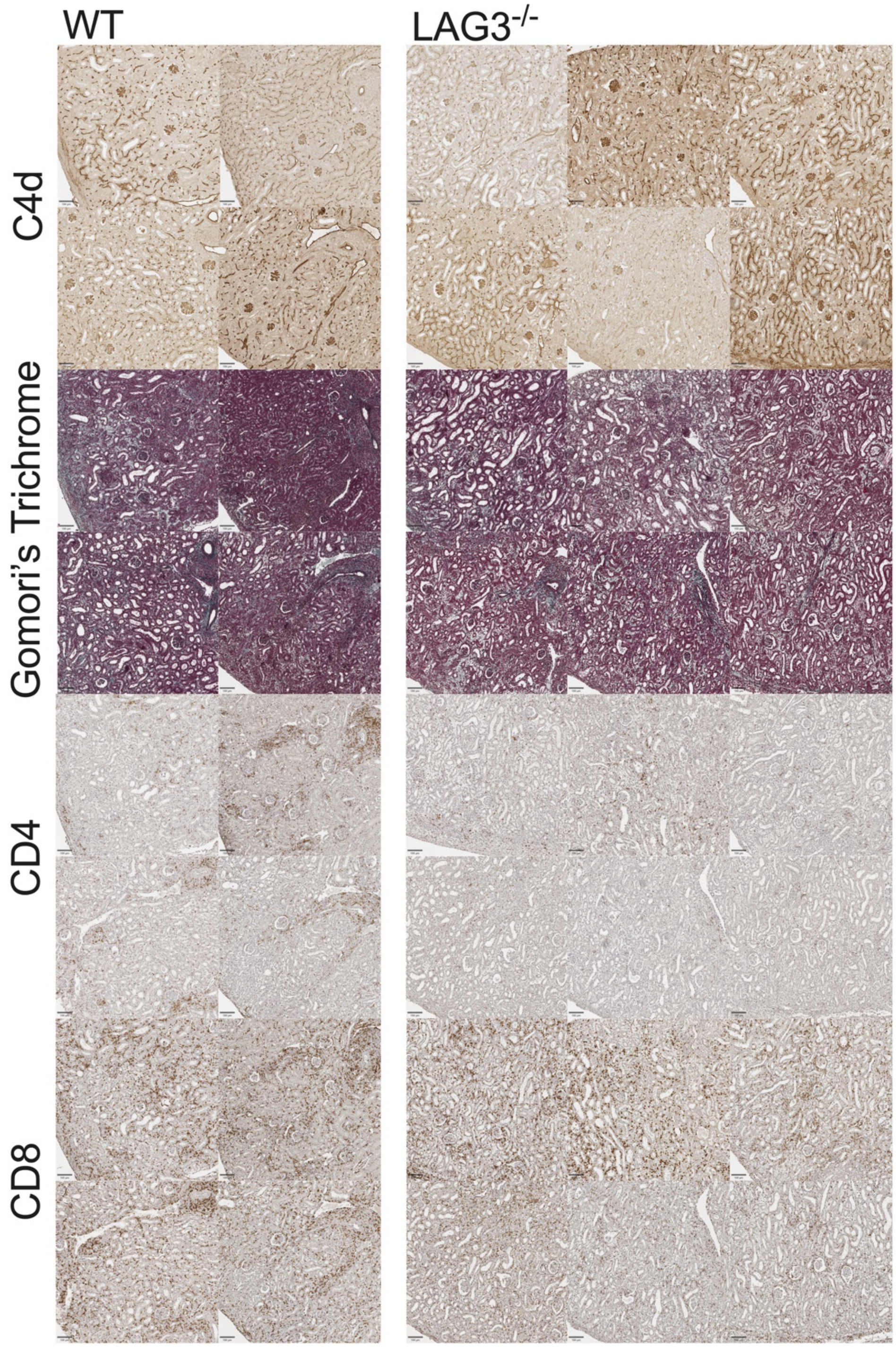

***Figure S3. Graft histology of WT and LAG3-/- renal allografts**. Renal allografts analyzed at the time of rejection (B6.LAG3$^{-/-}$) or on d. 14 posttransplant (B6.WT) by Trichrome C and immunoperoxidase staining for CD4, CD8 and complement component C4d. The photographs were taken at 200x and each animal is representative of an individual animal.*

Figure S4

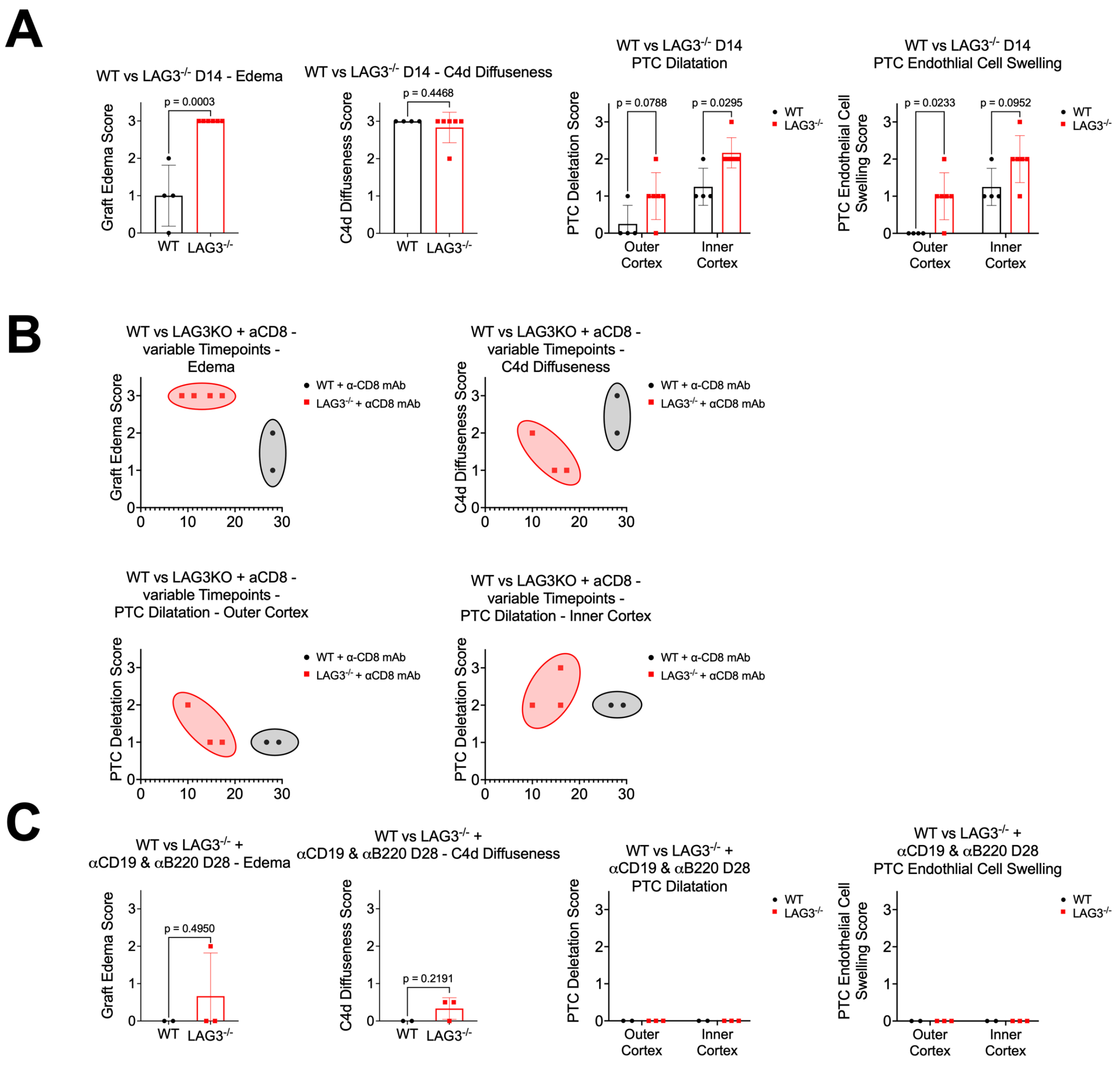

Figure S4. Quantification of histology scores of renal allografts*. Renal allografts analyzed at the various times posttransplant by a blinded pathologist. Immunohistology staining of C4d and Trichrome were used to determine states of edema, C4d diffuseness, and peritubular capillary (PTC) dilatation as described in the methods. Scoring criteria was is described in supplementary table 1 (Table S1).* ***A****. Quantification of histology scores in WT and LAG3-/- renal allograft recipients at d. 14 posttransplant.* ***B****. Quantification of histology scores in WT and LAG3-/- renal allograft recipients that received CD8 T cell depletion therapy at either rejection or at the d. 28-30, the experimental endpoint, posttransplant.* ***C****. Quantification of histology scores in WT and LAG3-/- renal allograft recipients that received B cell depletion therapy at d. 28 posttransplant. Each Data point represents and individual animal. Student's T tests were performed and p<0.05 were considered significant.*

Figure S5

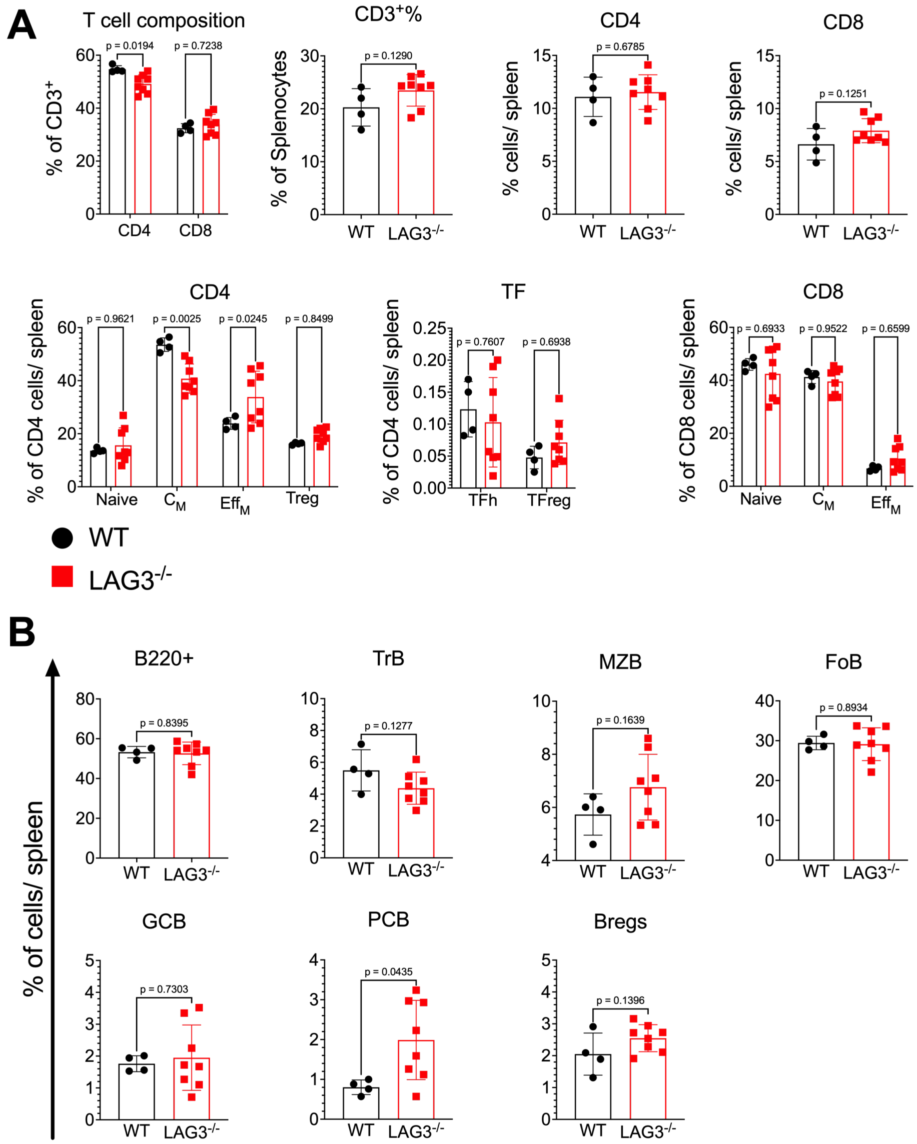

Figure S5. Recipient LAG3 deficiency enhances anti-donor immune responses. *Analyses of donor-reactive immunity in B6.WT and B6.LAG3$^{-/-}$ allograft recipients were performed at d. 10 posttranplant.* ***A.*** *The frequencies of spleen T cell subsets defined as follows: CD3 – CD3^{+}, CD4 – CD3^{+}CD4^{+}, CD8 - CD3^{+}CD8^{+}, Tregs - CD3^{+}CD4^{+} FoxP3^{+}, CD4 Naïve - CD3^{+}CD4^{+}CD62L^{hi}CD44^{lo}, CD4 C$_{M}$ - CD3^{+}CD4^{+}CD62L^{hi}CD44^{hi}, CD4 Eff$_{M}$ - CD3^{+}CD4^{+}CD62L^{lo}CD44^{hi}, CD8 Naïve - CD3^{+}CD8^{+}CD62L^{hi}CD44^{lo}, CD8 C$_{M}$ - CD3^{+}CD8^{+}CD62L^{hi}CD44^{hi}, CD8 Eff$_{M}$ - CD3^{+}CD8^{+}CD62L^{lo}CD44^{hi}, TFh - TCRb^{+}CD4^{+}FoxP3^{-}PD-1^{+}CXCR5^{+} and TFreg - TCRb^{+}CD4^{+}FoxP3^{+}PD-1^{+}CXCR5^{+}.* ***B.*** *The composition of splenic B cell defined as follows: B220 – B220^{+}, FoB - B220^{+}IgM^{int}CD21/35^{int}, MZB - B220^{+}IgM^{hi}CD21/35^{hi}, TrB - B220^{+}IgM^{hi}CD21/35^{lo}, Bregs - CD19^{+}CD1d^{hi}CD5^{+}, GCB - B220^{+}GL7^{+}CD38^{lo}, PCB - B220^{-} CD138^{hi}. Student's T tests were performed and $p<0.05$ were considered significant.*

Figure S6

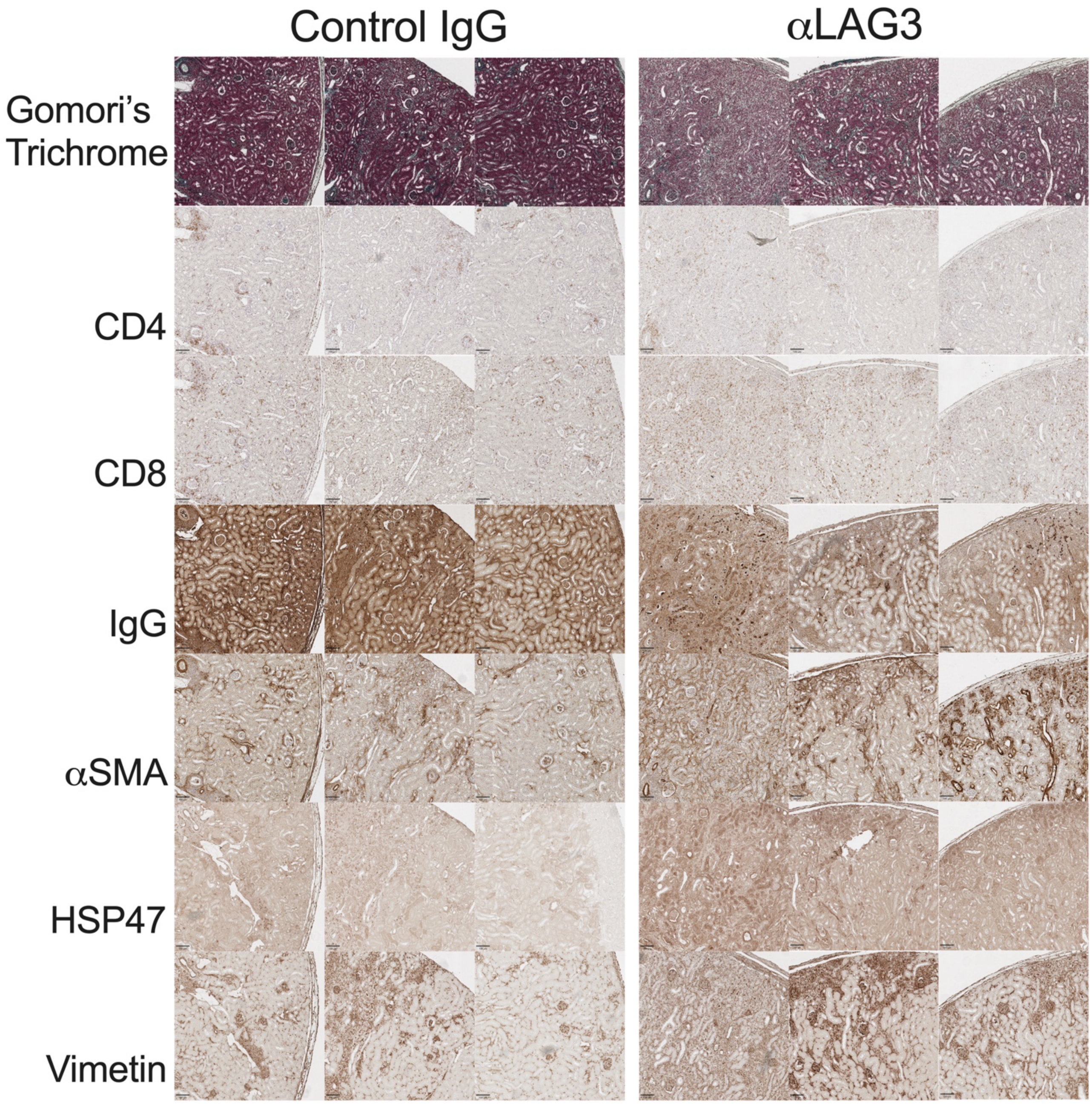

Figure S6. Graft histology of WT renal allografts treated with control IgG or anti-LAG3 mAbs. *Renal allografts analyzed at the time of rejection on d. 42 posttransplant by Trichrome C and immunoperoxidase staining for CD4, CD8, unconjugated IgG, smooth muscle actin (SMA), heat shock protein 47 (HSP47), and vimentin. The photographs were taken at 200x and each animal is representative of an individual animal.*

Figure S7

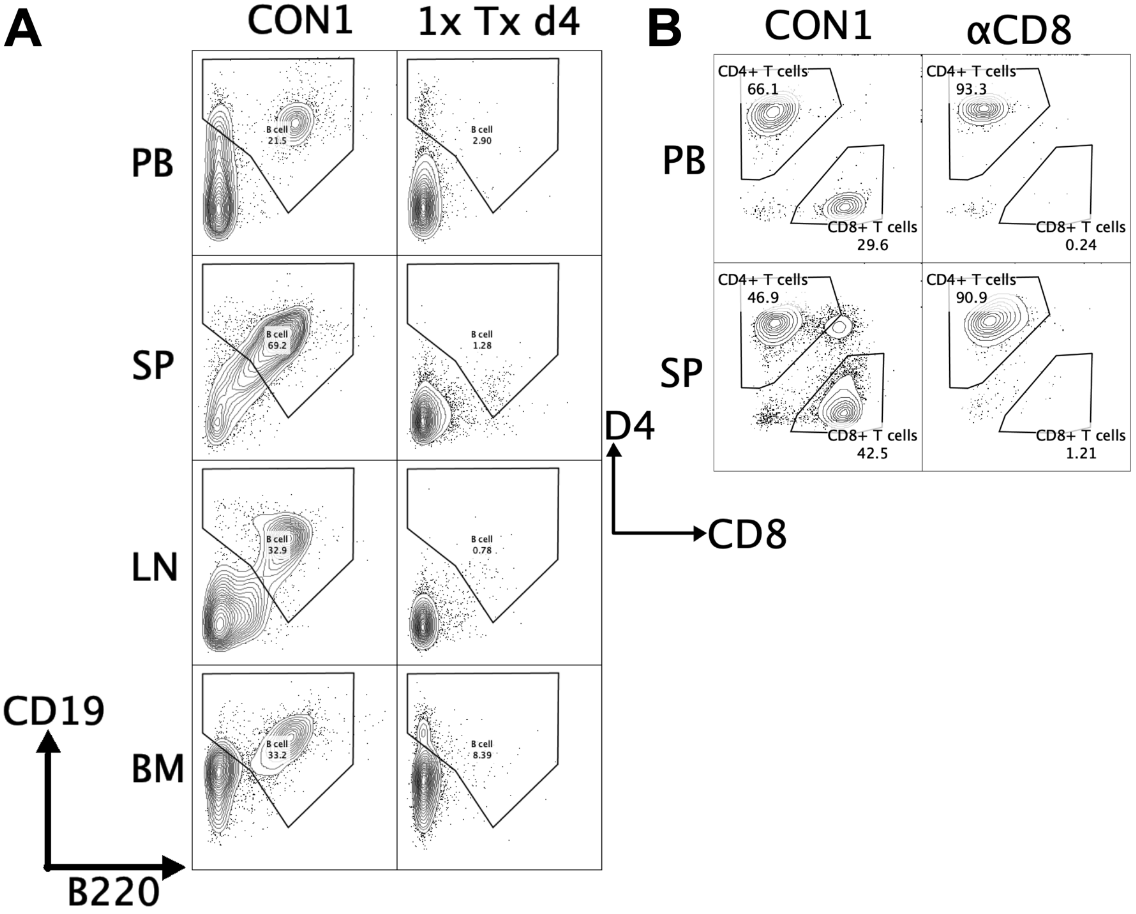

***Figure S7. Confirmation of lymphocyte depletion strategies**. **A**.* *B cells were depleted in B6.WT or B6.LAG3$^{-/-}$ recipients after transplantation of C3H renal allografts. Peripheral blood (PB), spleen (SP), inguinal lymph nodes (LN) and bone marrow (BM) were recovered on d4 and analyzed for the presence of CD19^{+} and B220^{+} B cells.* ***B**.* *CD8^{+} T cells were depleted in B6.WT or B6.LAG3$^{-/-}$ recipients after transplantation of C3H renal allografts. Peripheral blood (PB) and spleen (SP), were recovered on d4 and analyzed for the presence of CD4^{+} and CD8^{+} T cells.*

Figure S8

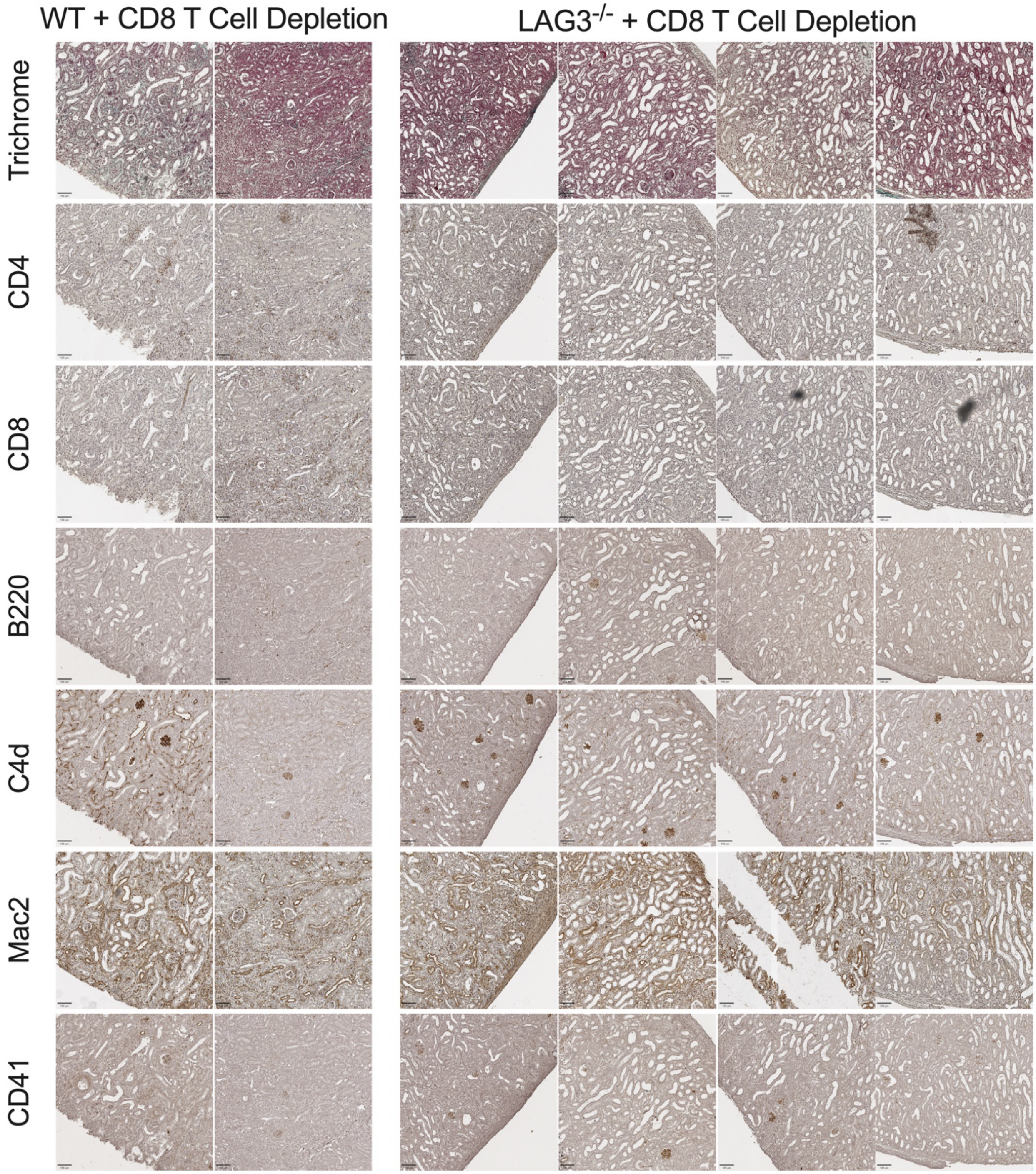

Figure S8. Graft histology of WT or LAG3$^{-/-}$ renal allografts treated with depleting CD8 mAbs. *Renal allografts analyzed at the time of rejection on d. 14 or 28-30 posttransplant by Trichrome C and immunoperoxidase staining for CD4, CD8, B220, complement component C4d, Mac and CD41. The photographs were taken at 200x and each animal is representative of an individual animal.*

Figure S9

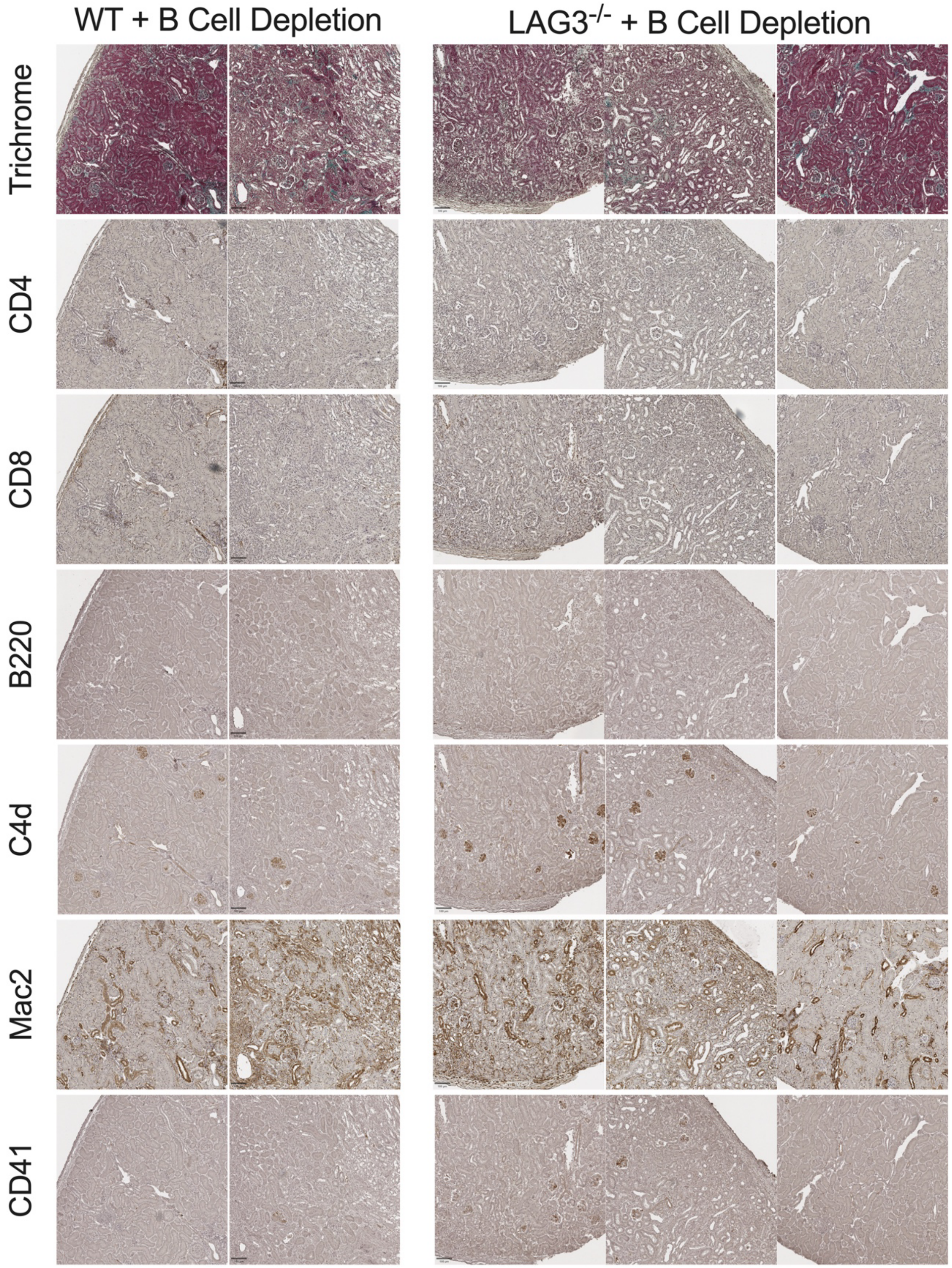

Figure S9. Graft histology of WT or LAG3$^{-/-}$ renal allografts treated with depleting CD19 and B220 mAbs. *Renal allografts analyzed on d. 28 posttransplant by Trichrome C and immunoperoxidase staining for CD4, CD8, B220, complement component C4d, Mac and CD41. The photographs were taken at 200x and each animal is representative of an individual animal.*

Figure S10

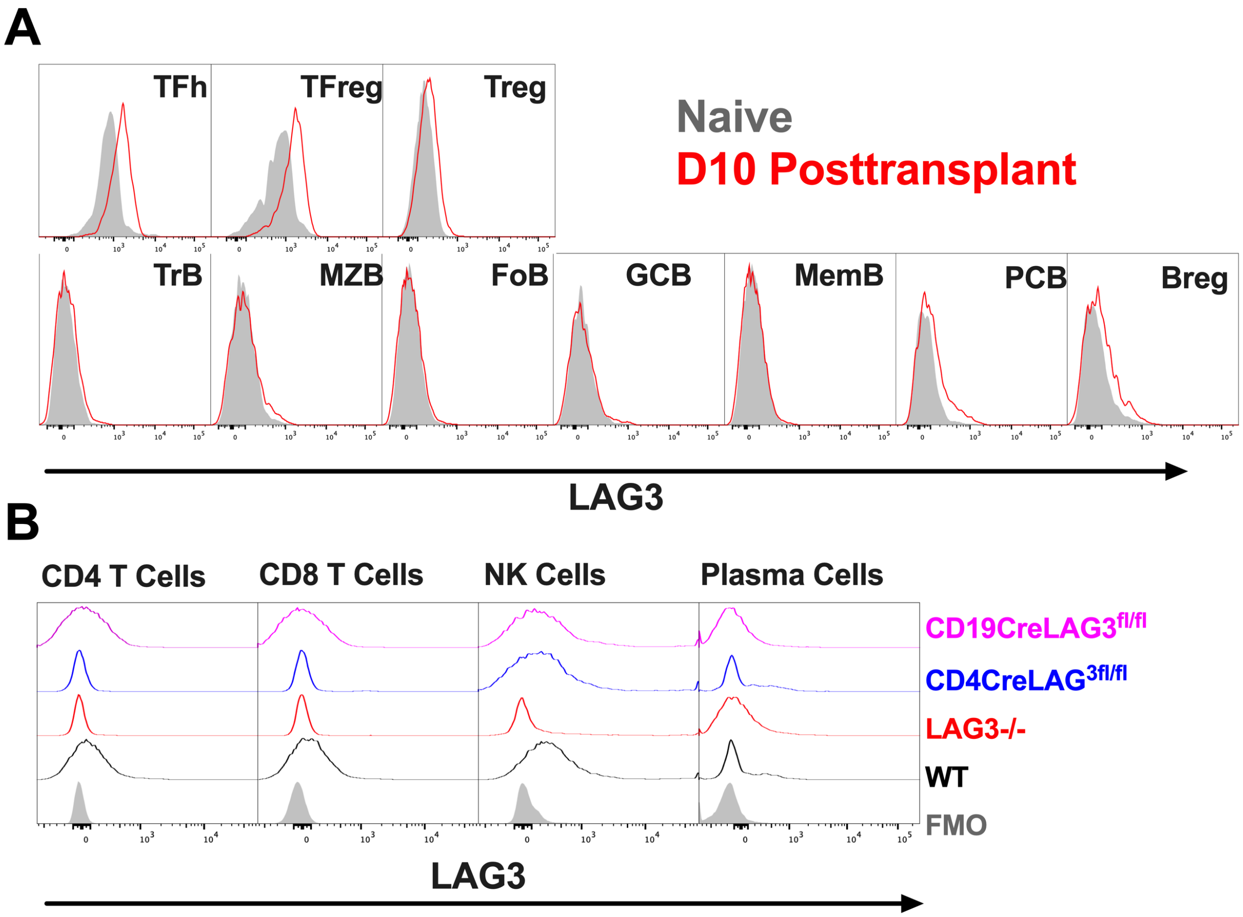

Figure S10. Flow cytometric analysis of LAG3 expression in various immune cells. A. *Analysis of LAG3 expression in follicular helper T cells (Tfh), follicular regulatory T cells (Tfreg), regulatory T cells (Treg), transitional B cells (TrB), marginal zone B cells (MZB), follicular B cells (FoB), germinal center B cells (GCB), memory B cells (MemB), plasma cells (PCB) and regulatory B cells (Breg) from splenocytes of naïve mice (gray-filled), and WT mice at d. 14 posttransplant (red).* ***B***. *Confirmation of LAG3 expression and conditional expression in WT (black), LAG3$^{-/-}$ (red), CD4CreLAG3$^{fl/fl}$ (blue), and CD19CreLAG3$^{fl/fl}$ (pink) mice.*

Figure S11

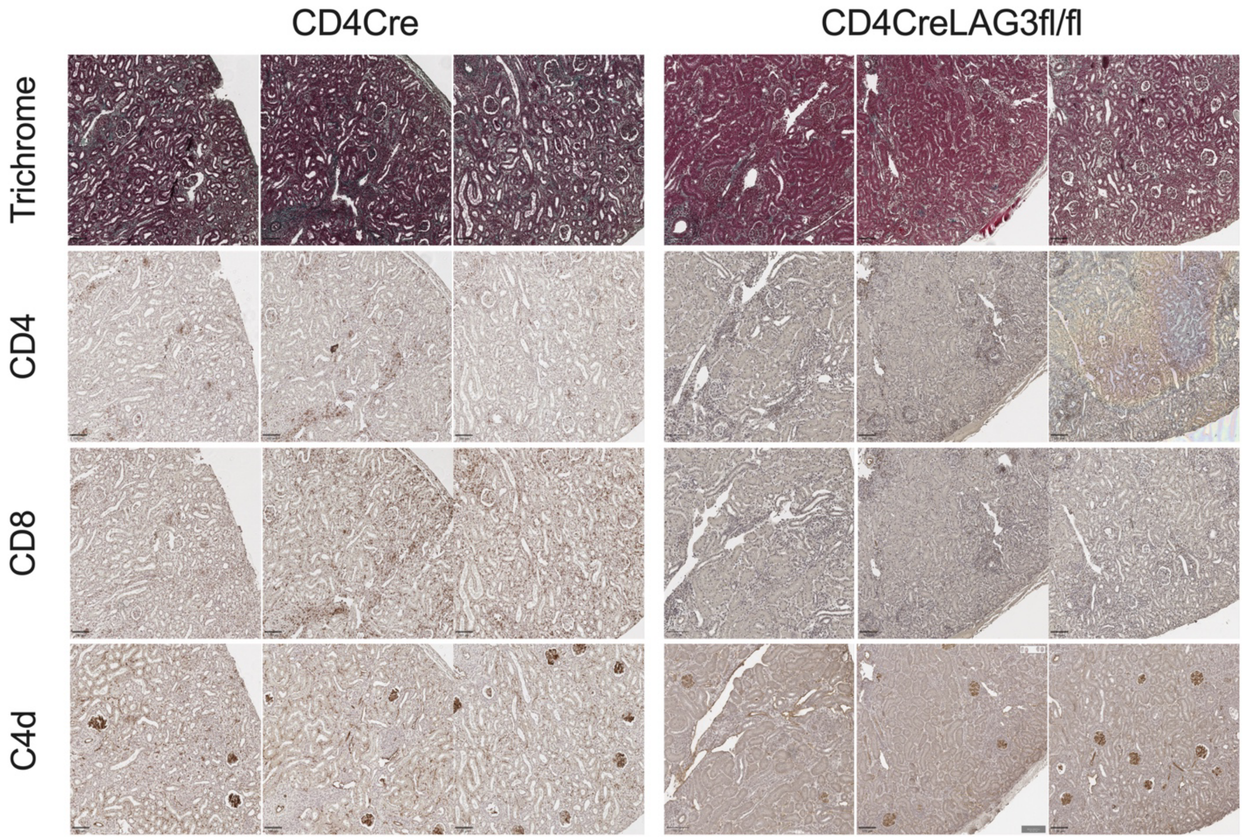

Figure S11. Graft histology of CD4Cre or CD4CreLAG3$^{fl/fl}$ renal allografts. *Renal allografts analyzed on d. 28 posttransplant by Trichrome C and immunoperoxidase staining for CD4, CD8, and complement component C4d. The photographs were taken at 200x and each animal is representative of an individual animal.*

Figure S12

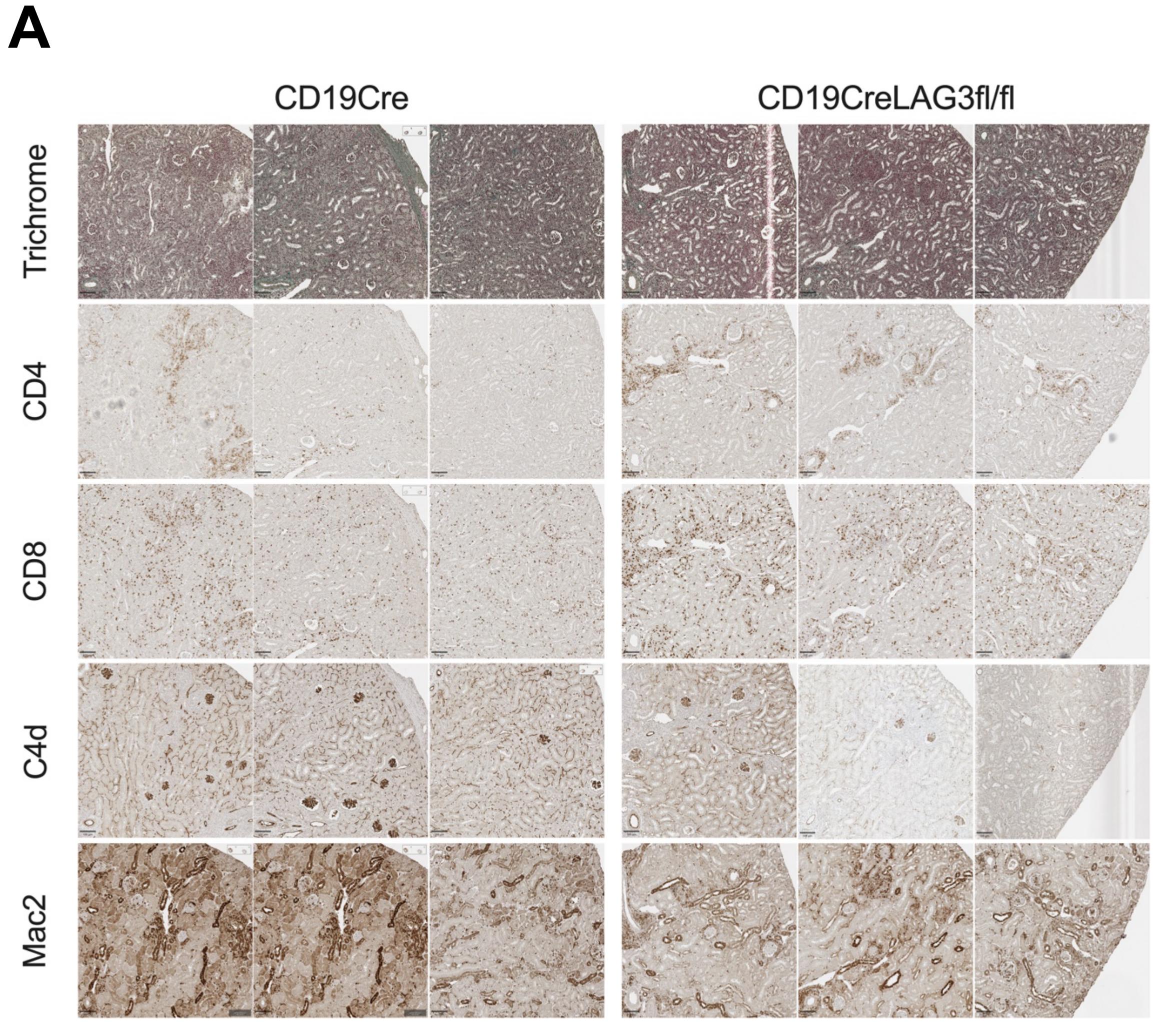

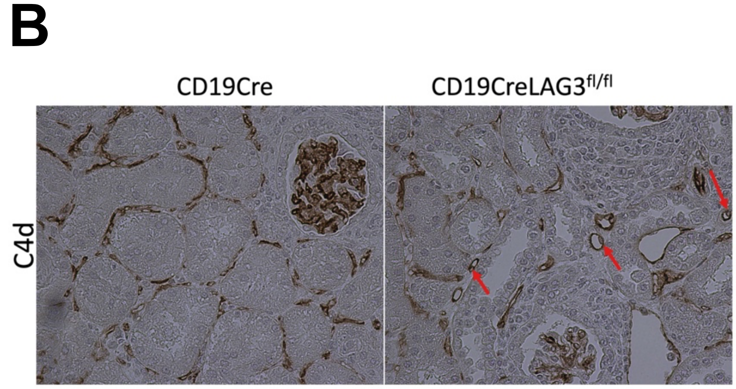

Figure S12. Graft histology of CD19Cre or CD19CreLAG3$^{fl/fl}$ renal allografts. A. *Renal allografts analyzed on d. 28 posttransplant by Trichrome C and immunoperoxidase staining for CD4, CD8, and complement component C4d. The photographs were taken at 200x and each animal is representative of an individual animal.* ***B****. CD19Cre (left) or CD19CreLAG3$^{fl/fl}$ (right) were transplanted with C3H kidney allografts that were recovered on d30 posttransplant and stained for C4d. Red arrows indicate swollen endothelial cells indicating antibody mediated rejection.*

Figure S13

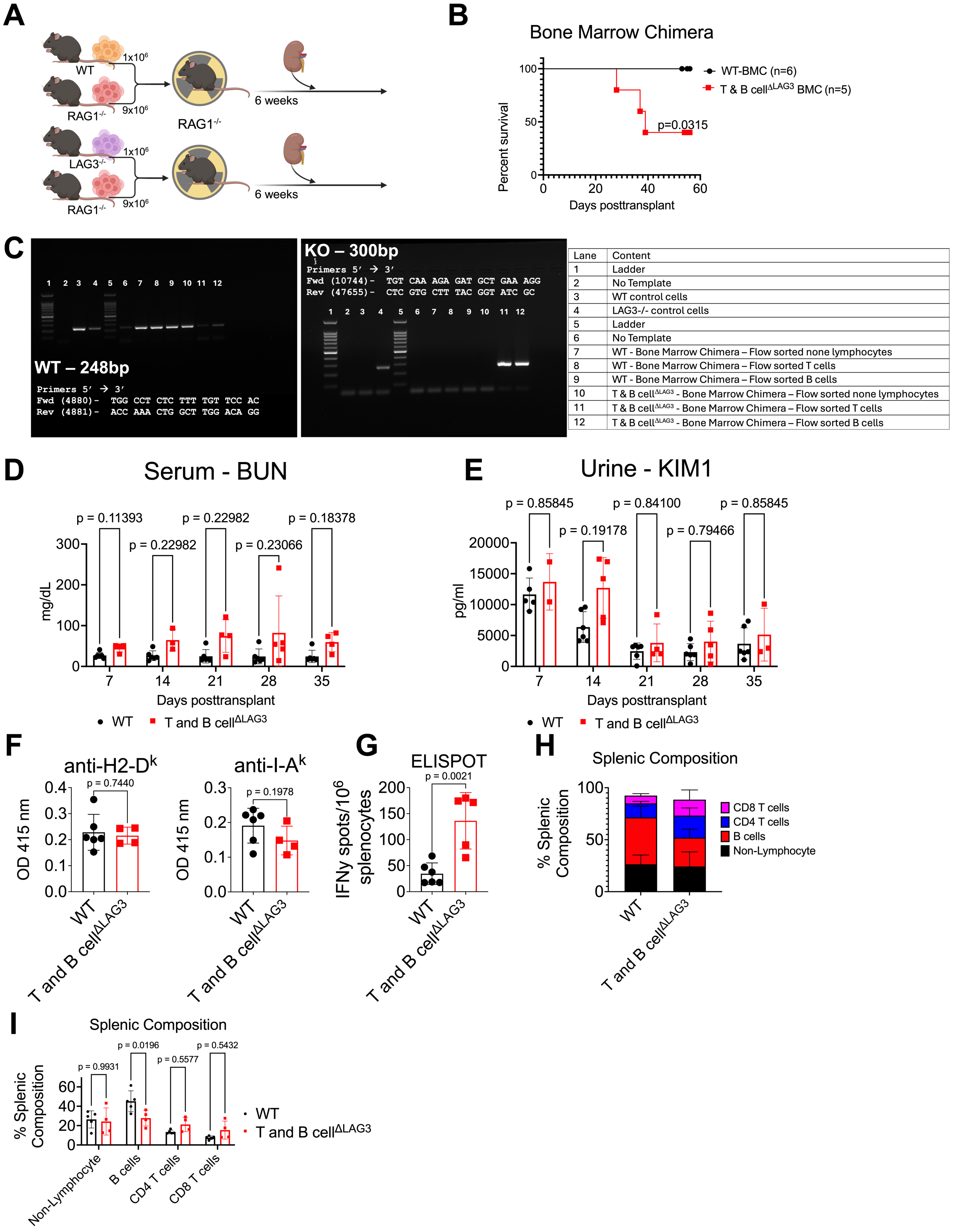

Lane	Content
1	Ladder
2	No Template
3	WT control cells
4	LAG3-/- control cells
5	Ladder
6	No Template
7	WT - Bone Marrow Chimera – Flow sorted none lymphocytes
8	WT - Bone Marrow Chimera – Flow sorted T cells
9	WT - Bone Marrow Chimera – Flow sorted B cells
10	T & B cellΔLAG3 - Bone Marrow Chimera – Flow sorted none lymphocytes
11	T & B cellΔLAG3 - Bone Marrow Chimera – Flow sorted T cells
12	T & B cellΔLAG3 - Bone Marrow Chimera – Flow sorted B cells

***Figure S13. Loss of LAG3 regulation on T and B lymphocytes is sufficient to precipitate kidney allograft rejection**. **A**. Bone marrow cells from WT and $RAG1^{-/-}$ ($1x10^6$ and $9x10^6$ of each, respectively), or $LAG3^{-/-}$ and $RAG1^{-/-}$ ($1x10^6$ and $9x10^6$ of each, respectively), were adoptively transferred into lethally irradiated $RAG1^{-/-}$ mice. 6 weeks after adoptive transfer bone marrow chimeric mice were transplanted with C3H renal allografts. **B**. Survival of renal allografts (n=5-6/group). **C**. PCR confirmation of T and B cell double conditional LAG3 mouse generation from flow sorted splenocytes. **D**. Serum kidney injury marker, Blood Urea Nitrogen (BUN) levels from chimeric kidney allograft recipients. **E**. Kidney injury marker KIM1 from the urine of chimeric kidney allograft recipients. **F**. Serum levels of IgG against donor MHC-I (H2-Dk) and MHC9-II (I-Ak) in control or T and B cell LAG3-/- chimeric kidney transplant recipients from d21 posttransplant. **G**. The frequencies of donor reactive IFNγ secreting splenocytes on d. 56 posttransplant. **H&I**. Splenic composition of chimeric mice following transplantation. Statistical analysis of allograft survival was measured using Mantel-Cox log-rank test and for other analyses student's T tests were performed and $p<0.05$ were considered significant*

Figure S14

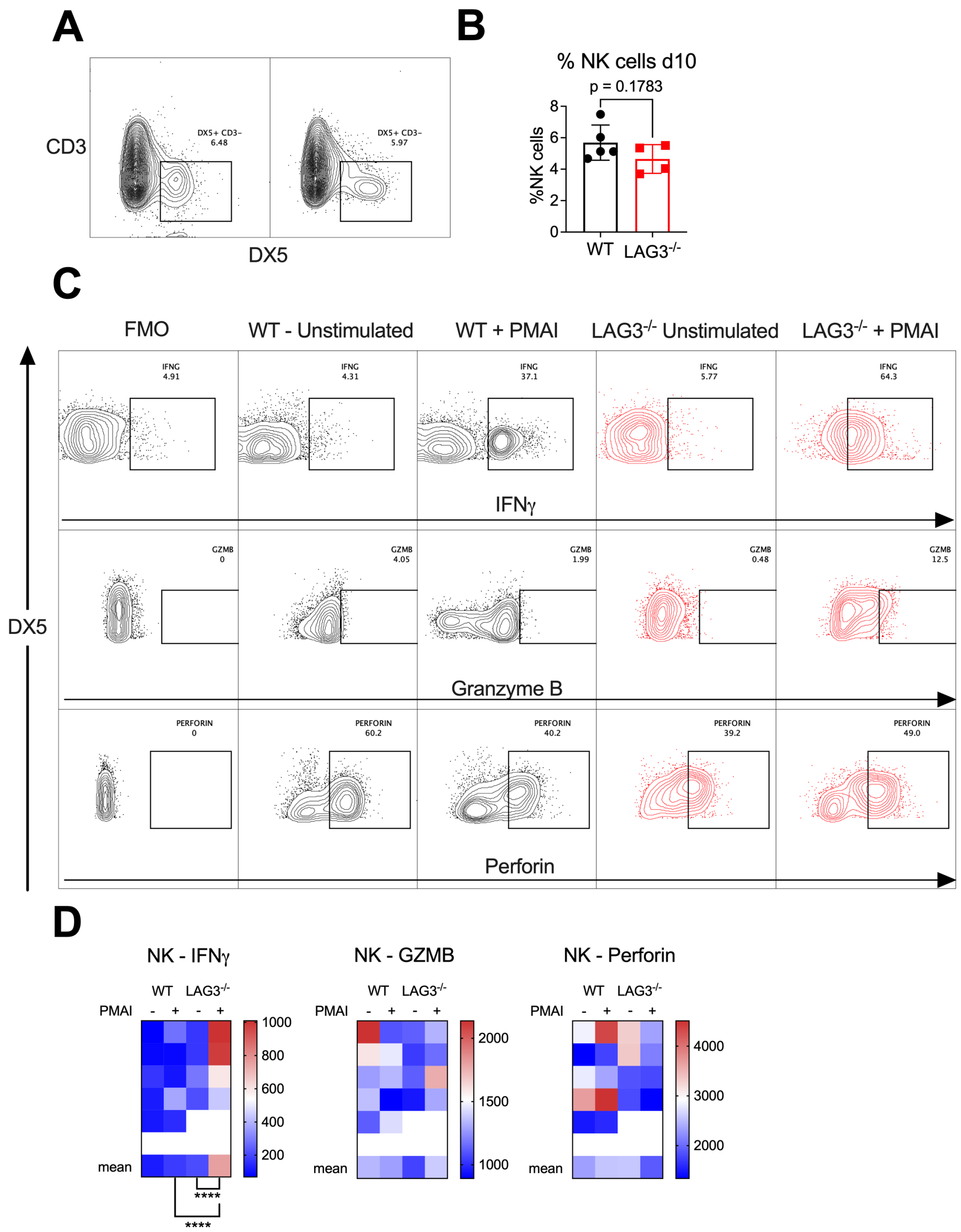

Figure S14. LAG3 regulation of graft infiltrating NK cell responses. A. *Representative gating of graft NK cells. CD45+ Live graft infiltrating cells taken at d10 posttransplant from WT (left) and $LAG3^{-/-}$ kidney transplant recipients, demonstrating gating of NK cells ($DX5^{+}CD3^{-}$).* ***B****. Quantification of the frequencies of graft infiltrating NK cells at d. 10 posttransplant in WT and $LAG3^{-/-}$ kidney transplant recipients.* ***C****. Representative contour plots demonstrating cytokine staining (IFNγ – top, Granzyme B -middle, Perforin - bottom) of graft infiltrating NK cells before and after 4 hours of stimulation with PMAI.* ***D*** *. Heatmaps of MFIs of graft infiltrating NK cell staining for IFNg, Granzyme B (GZMB) and Perforin from WT or $LAG3^{-/-}$ mice on d.10 posttransplant. For heatmaps individual mice are shown above the*

*indicated mean MFI values. Student's T tests were performed and $p<0.05$ were considered significant. Blank – not significant $P > 0.05$, * = $P \le 0.05$, ** = $P \le 0.01$, *** = $P \le 0.001$, **** = $P \le 0.0001$.*

Figure S15

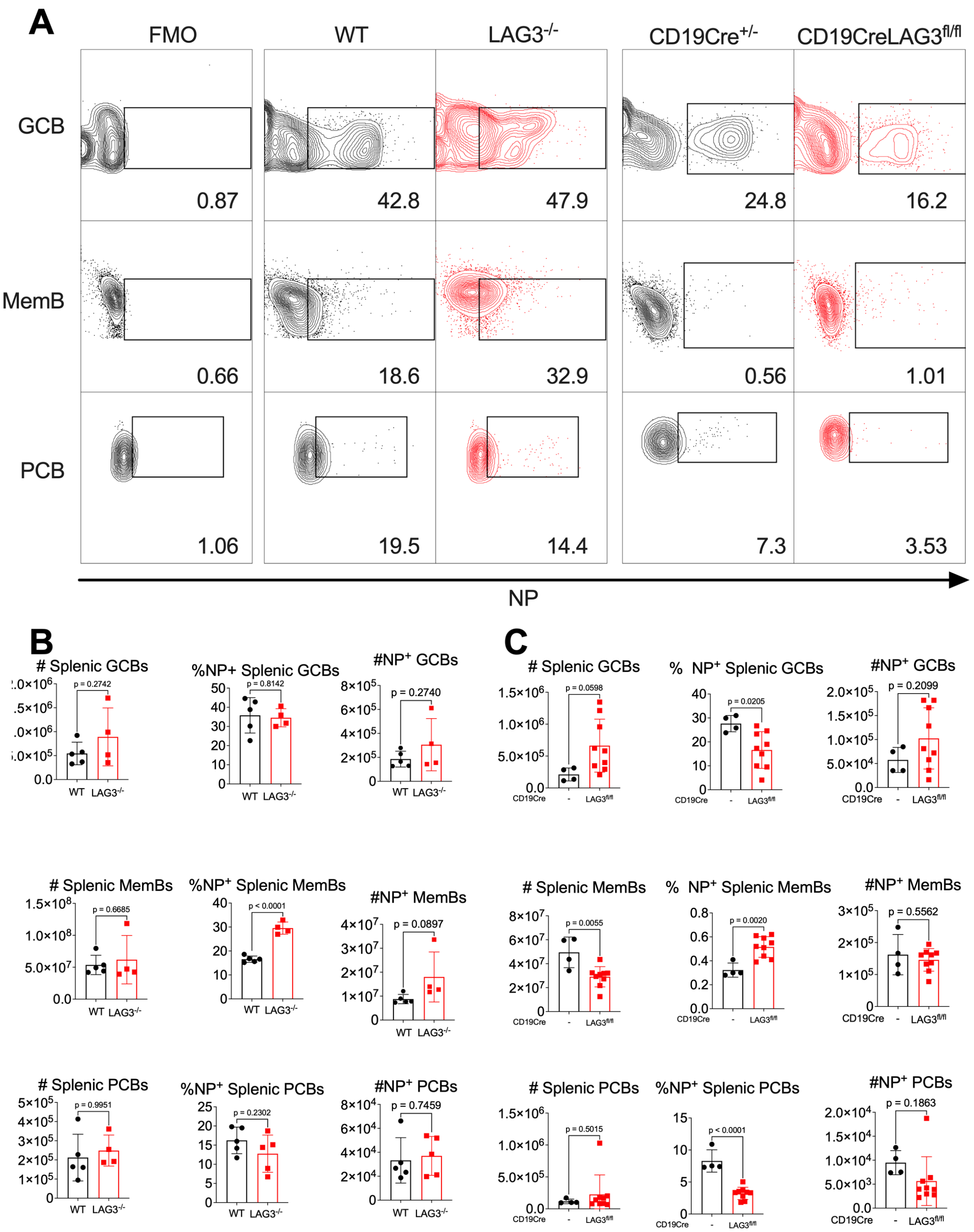

Figure S15. LAG3 regulation of B cell responses following immunization with T-dependent antigen NP-KLH. A*. Representative contour plots demonstrating NP binding of splenic B cell populations on d21 following immunization with NP-KLH.* ***B****. Quantification of numbers of total and frequencies and numbers of NP reactive splenic germinal center B cells (GCBs), memory B cells (MemB) and plasma cells (PCB) on day 21 following immunization of WT and LAG3$^{-/-}$ mice with NP-KLH.* ***C****. Quantification of numbers of total and frequencies of NP reactive splenic germinal center B cells (GCBs), memory B cells (MemB) and plasma cells (PCB) on day 21 following immunization of CD19Cre and CD19CreLAG3$^{fl/fl}$ mice with NP-KLH.*

Figure S16.

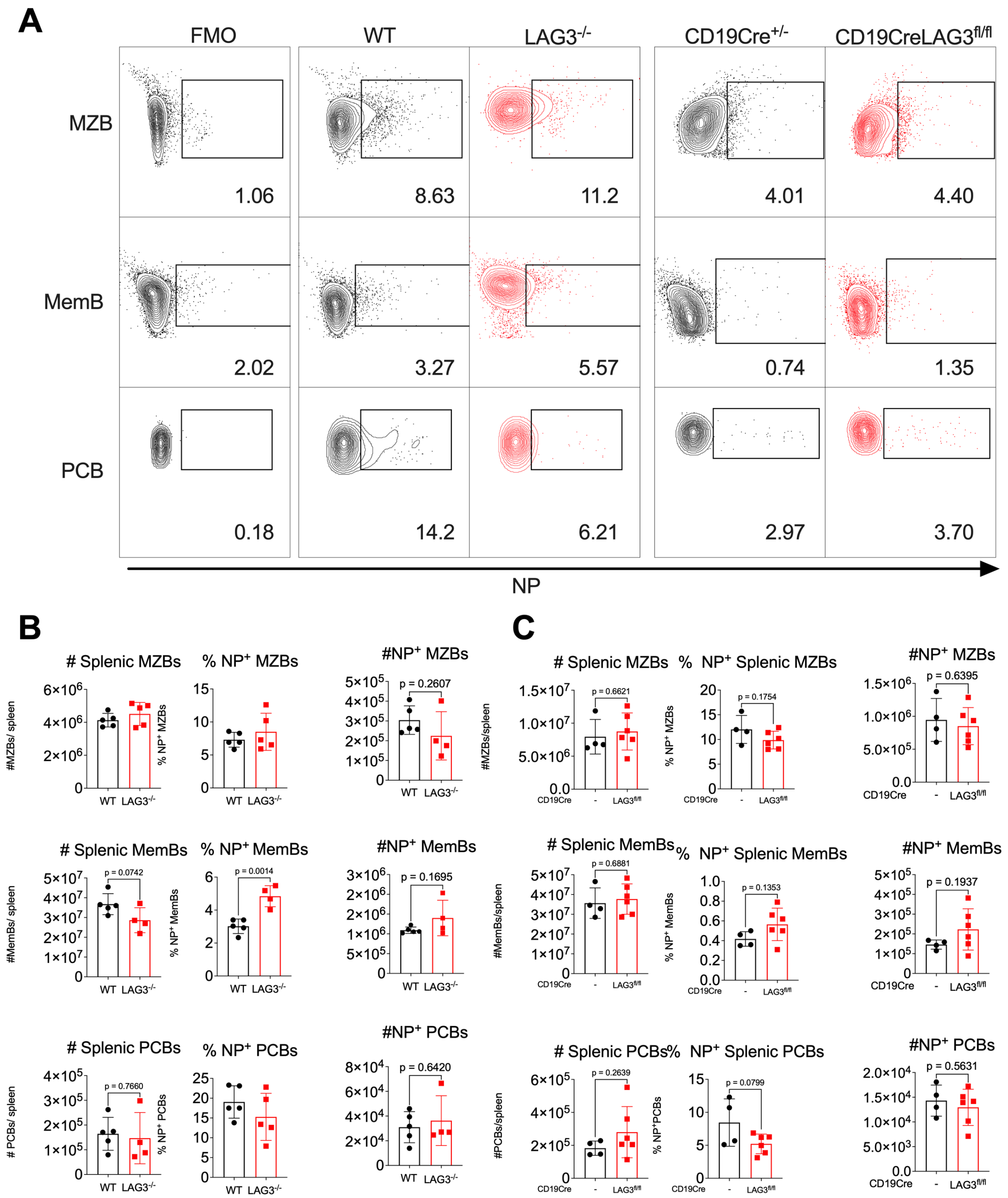

Figure S16. LAG3 regulation of B cell responses following immunization with T-independent antigen NP-AECM-FICOLL. A*. Representative contour plots demonstrating NP binding of splenic B cell populations on d21 following immunization with NP-AECM-FICOLL.* ***B****. Quantification of numbers of total and frequencies and numbers of NP reactive splenic germinal center B cells (GCBs), memory B cells (MemB) and plasma cells (PCB) on day 21 following immunization of WT and LAG3$^{-/-}$ mice with NP-AECM-FICOLL.* ***C****. Quantification of numbers of total and frequencies of NP reactive splenic marginal zone B cells (MZB), memory B cells (MemB) and plasma cells (PCB) on day 21 following immunization of CD19Cre and CD19CreLAG3$^{fl/fl}$ mice with NP-AECM-FICOLL.*

Figure S17.

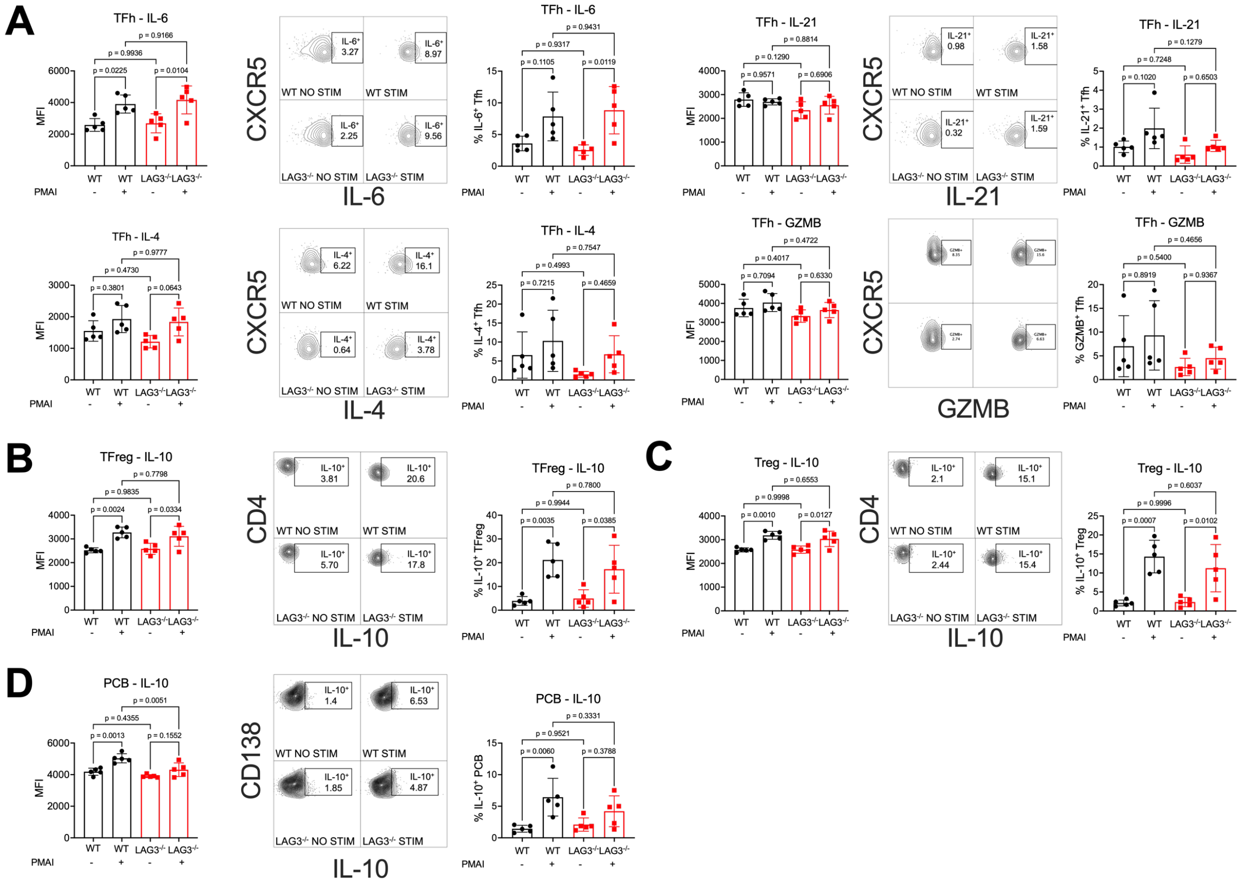

Figure S17. LAG3 regulation of cytokine production by immune cell subsets following immunization with T-dependent antigen NP-KLH. A*. Quantification of MFIs from flow cytometric staining for IL-6, IL-21, IL-4 and granzyme B of splenic Tfh cells at d.10 following immunization of WT and LAG3$^{-/-}$ mice with NP-KLH, representative contour plots of cytokine staining before and after stimulation with PMAI, frequencies of cytokine positive Tfh cells from NP-KLH immunized WT and LAG3$^{-/-}$ mice.* ***B-D****. Quantification of MFI from flow cytometric staining for IL-10 in splenic Tfreg (****B****), Treg (****C****), and plasma cells (PCB) (****D****) at d.10 following immunization of WT and LAG3$^{-/-}$ mice with NP-KLH, representative contour plots of cytokine staining before and after stimulation with PMAI, frequencies of cytokine positive Tfreg (****B****), Treg (****C****), and plasma cells (****D****) from NP-KLH immunized WT and LAG3$^{-/-}$ mice.*

1. Zhang Z, Schlachta C, Duff J, Stiller C, Grant D, Zhong R. Improved techniques for kidney transplantation in mice. *Microsurgery*. 1995;16:103-109.
2. Han WR, Murray-Segal LJ, Mottram PL. Modified technique for kidney transplantation in mice. *Microsurgery*. 1999;19:272-274.
3. Murata K, Fox-Talbot K, Qian Z, Takahashi K, Stahl GL, Baldwin WM, 3rd, Wasowska BA. Synergistic deposition of C4d by complement-activating and non-activating antibodies in cardiac transplants. *Am J Transplant*. 2007;7:2605-2614.
4. Gorbacheva V, Fan R, Miyairi S, Fairchild RL, Baldwin WM, 3rd, Valujskikh A. Autoantibodies against DNA topoisomerase I promote renal allograft rejection by increasing alloreactive T cell responses. *Am J Transplant*. 2023;23:1307-1318. doi: 10.1016/j.ajt.2023.03.027
5. Lucas CL, Workman CJ, Beyaz S, LoCascio S, Zhao G, Vignali DA, Sykes M. LAG-3, TGF-beta, and cell-intrinsic PD-1 inhibitory pathways contribute to CD8 but not CD4 T-cell tolerance induced by allogeneic BMT with anti-CD40L. *Blood*. 2011;117:5532-5540. doi: 10.1182/blood-2010-11-318675
6. Workman CJ, Rice DS, Dugger KJ, Kurschner C, Vignali DA. Phenotypic analysis of the murine CD4-related glycoprotein, CD223 (LAG-3). *Eur J Immunol*. 2002;32:2255-2263. doi: 10.1002/1521-4141(200208)32:8<2255::AID-IMMU2255>3.0.CO;2-A
7. Gorbacheva V, Fan R, Fairchild RL, Baldwin WM, 3rd, Valujskikh A. Memory CD4 T Cells Induce Antibody-Mediated Rejection of Renal Allografts. *J Am Soc Nephrol*. 2016. doi: 10.1681/ASN.2015080848
8. Ayasoufi K, Kohei N, Nicosia M, Fan R, Farr GW, McGuirk PR, Pelletier MF, Fairchild RL, Valujskikh A. Aquaporin 4 blockade improves survival of murine heart allografts subjected to prolonged cold ischemia. *Am J Transplant*. 2018;18:1238-1246. doi: 10.1111/ajt.14624
9. Rabant M, Gorbacheva V, Fan R, Yu H, Valujskikh A. CD40-independent help by memory CD4 T cells induces pathogenic alloantibody but does not lead to long-lasting humoral immunity. *Am J Transplant*. 2013;13:2831-2841. doi: 10.1111/ajt.12432
10. Sicard A, Phares TW, Yu H, Fan R, Baldwin WM, 3rd, Fairchild RL, Valujskikh A. The spleen is the major source of antidonor antibody-secreting cells in murine heart allograft recipients. *Am J Transplant*. 2012;12:1708-1719. doi: 10.1111/j.1600-6143.2012.04009.x
11. Nicosia M, Miyairi S, Beavers A, Farr GW, McGuirk PR, Pelletier MF, Valujskikh A. Aquaporin 4 inhibition alters chemokine receptor expression and T cell trafficking. *Sci Rep*. 2019;9:7417. doi: 10.1038/s41598-019-43884-2
12. Ayasoufi K, Fan R, Fairchild RL, Valujskikh A. CD4 T Cell Help via B Cells Is Required for Lymphopenia-Induced CD8 T Cell Proliferation. *J Immunol*. 2016;196:3180-3190. doi: 10.4049/jimmunol.1501435
13. Gorbacheva V, Fan R, Li X, Valujskikh A. Interleukin-17 promotes early allograft inflammation. *Am J Pathol*. 2010;177:1265-1273. doi: 10.2353/ajpath.2010.091106
14. Zhang Q, Chen Y, Fairchild RL, Heeger PS, Valujskikh A. Lymphoid sequestration of alloreactive memory CD4 T cells promotes cardiac allograft survival. *J Immunol*. 2006;176:770-777.
