# Supplementary material for: LAG3 regulates antibody responses in a murine model of kidney transplantation
Source: J Clin Invest. 2025 May 13;135(13):e172988. doi: 10.1172/JCI172988 (PMC12208549; doi:10.1172/JCI172988)

Unedited gel images

(172988-JCI-RG-RV-3)

Nicosia et al.

Full unedited gel for Figure S13C, left

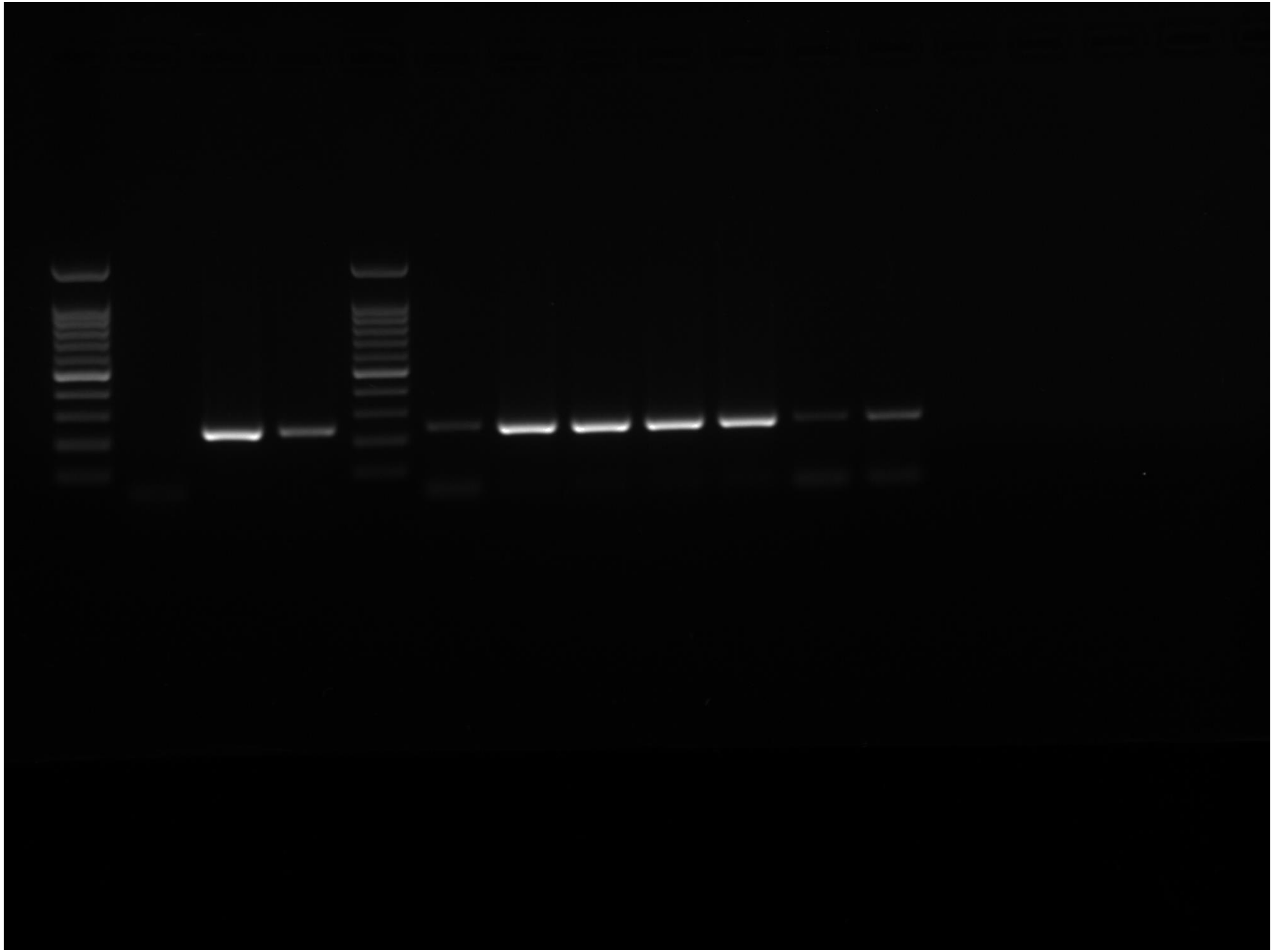

Full unedited gel for Figure S13C, right

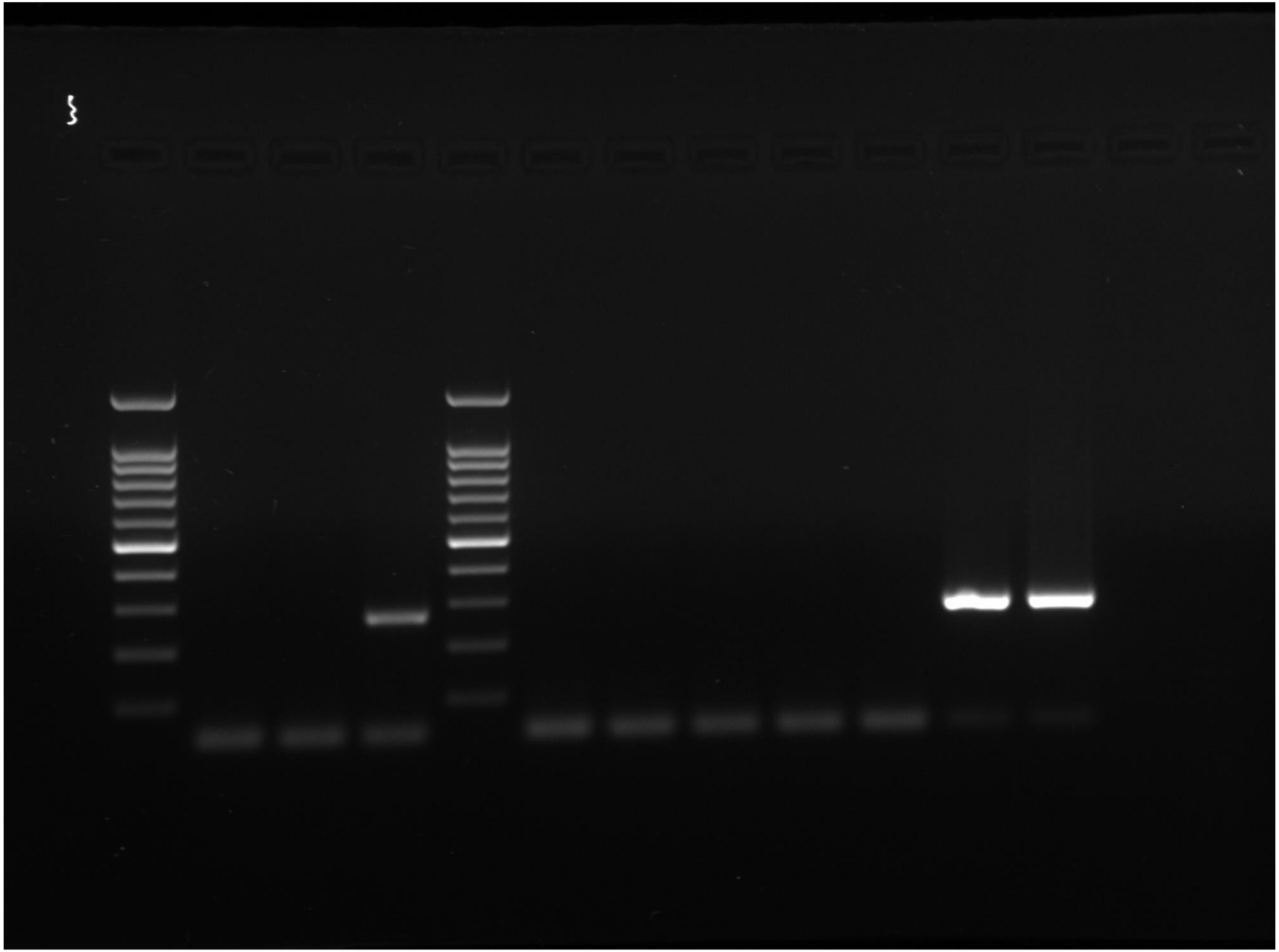

Supplement: Unedited blot and gel images [file jci-135-172988-s163.pdf]
